# Disorder policing to reduce crime: A systematic review

**DOI:** 10.1002/cl2.1050

**Published:** 2019-09-08

**Authors:** Anthony A. Braga, Brandon C. Welsh, Cory Schnell

**Affiliations:** ^1^ School of Criminology and Criminal Justice Northeastern University Boston Massachusetts; ^2^ Department of Criminology and Criminal Justice University of South Carolina Columbia South Carolina

## 1 PLAIN LANGUAGE SUMMARY

Policing disorder through community policing and problem‐solving policing is associated with reductions in crime, but aggressive, order maintenance approaches do not seem to generate crime reductions.

**What is the aim of this review?**

This Campbell systematic review examines the effects of disorder policing interventions on crime. The review summarizes evidence from 28 high‐quality studies (representing 30 independent tests), including nine randomized controlled trials. Most the studies come from the United States.

### 1.1 The review in brief

Disorderly conditions are seen as a precursor to more serious crime, fear of crime, and neighborhood decline. Policing disorder is associated with reductions in crime, but only when community and problem‐solving tactics are used. Aggressive, order maintenance based approaches do not seem to be effective.

### 1.2 What is this review about?

Policing social and physical disorderly conditions is rooted in the broken windows approach: disorder is a precursor to more serious crime, fear of crime, and neighborhood decline. Addressing disorder has become a central fixture of policing, especially in the United States. Yet, evaluations of the effectiveness of disorder policing strategies in controlling crime yield conflicting results.

Policing disorderly conditions can be divided into two main strategies: (a) order maintenance or zero tolerance policing, where police attempt to impose order through strict enforcement and (b) community policing and problem‐solving policing, where police attempt to produce order and reduce crime through cooperation with community members and by addressing specific recurring problems.

This review examined the effects of disorder policing strategies compared to traditional law enforcement actions (e.g., regular levels of patrol) on the rates of crime, including property crime, violent crime, and disorder/drug crime. This review also examined whether policing disorder actions at specific locations result in crime displacement (i.e., crime moving around the corner) or diffusion of crime control benefits (i.e., crime reduction in surrounding areas).

#### 1.2.1 What studies are included?

A total of 28 disorder policing studies (representing 30 independent tests) met the criteria to be included in this review. The studies spanned the period from 1985 to 2012, and were mostly carried out in the United States. All of the studies used high‐quality designs to evaluate the impact of the intervention; nine were randomized controlled trials. Twelve tests were completed in large cities with more than 500,000 residents, nine tests were completed in medium‐sized cities with between 200,000 and 500,000 residents, and the other nine tests were completed in smaller cities with <200,000 residents. All of the tests were carried out in specific geographical settings, including small places (e.g., crime hot spots and problem buildings), smaller police‐defined areas (e.g., patrol beats), neighborhoods and selected stretches of highways, and larger police‐defined areas (e.g., precincts and divisions).

### 1.3 What are the main findings of this review?

*Do policing interventions focused on disorderly conditions reduce crime?*

Yes, in addition to an overall reduction in crime, there is a reduction in property crime, violent crime, and disorder/drug crime when disorder policing interventions are implemented.

*Do policing interventions focused on disorder result in crime being displaced or crime control benefits being diffused to surrounding areas?*

Disorder policing interventions are associated with diffusion of crime control benefits in areas surrounding targeted locations. This conclusion is based on 15 tests that measured displacement or diffusion effects.

*Of the two main strategies used in policing disorder, is one more effective than the other?*

Yes, policing disorder through community and problem‐solving is associated with reductions in crime. Aggressive, order maintenance approaches do not seem to generate crime reductions.

### 1.4 What do the findings of this review mean?

The types of strategies used by police departments to address disorderly conditions seem to matter in controlling crime, and this holds important implications for police–community relations, justice, and crime prevention. Further research is needed to understand the key programmatic elements that maximize the capacity of these strategies to prevent crime.

### 1.5 How up‐to‐date is this review?

This review includes studies completed before 2013. This Campbell review was published in September 2019.

## 2 BRIEF ABSTRACT

### 2.1 Background

Crime policy scholars and practitioners have argued for years that when police address social and physical disorder in neighborhoods they can prevent serious crime, yet evaluations of the crime control effectiveness of disorder policing strategies yield conflicting results. This review reports the results of a systematic review and meta‐analysis of the effects of disorder policing on crime.

### 2.2 Objectives

To assess the effects of disorder policing interventions on crime.

### 2.3 Search methods

Multiple search strategies were used to identify eligible studies. These strategies included a keyword search of online abstract databases, hand searches of relevant journals, consultation with policing experts, and searches of bibliographies of past narrative, empirical, and systematic reviews of police crime prevention efforts.

### 2.4 Selection criteria

Suitable interventions included tactics such as aggressive disorder enforcement as well as community and problem‐oriented policing explicitly designed to control crime by addressing disorder. Studies that used randomized experimental or quasiexperimental designs were selected.

### 2.5 Data collection and analysis

Twenty‐eight studies containing 30 tests of disorder policing interventions were identified. A formal meta‐analysis was conducted to determine the crime prevention effects of the eligible studies.

### 2.6 Results

Policing disorder strategies are associated with an overall statistically significant, modest crime reduction effect. Community and problem‐solving interventions generated crime reductions while aggressive order maintenance strategies did not.

### 2.7 Authors' conclusions

The types of strategies used by police departments to address disorder seem to matter in controlling crime, and this holds important implications for police–community relations, justice, and crime prevention.

## 3 EXECUTIVE SUMMARY

### 3.1 Background

Crime policy scholars and practitioners have argued for years that when police address social and physical disorder in neighborhoods they can prevent serious crime, yet evaluations of the crime control effectiveness of disorder policing strategies yield conflicting results. This review reports on the results of the first systematic review and meta‐analysis of the effects of disorder policing on crime.

### 3.2 Objectives

To assess the effects of disorder policing interventions on crime. The review also examined whether policing disorder actions at specific locations result in crime displacement (i.e., crime moving around the corner) or diffusion of crime control benefits (i.e., crime reduction in surrounding areas).

### 3.3 Search methods

A keyword search was performed on 15 online abstract databases. Bibliographies of past narrative and empirical reviews of literature that examined the effectiveness of police crime control programs were reviewed and forward searches for works that cited seminal disorder policing studies were performed. Bibliographies of past completed Campbell systematic reviews of police crime prevention efforts and hand searches of leading journals in the field were performed. Experts in the field were consulted and relevant citations were obtained. This review includes eligible studies completed before 2013.

### 3.4 Selection criteria

To be eligible for this review, interventions used to control disorder were limited to police enforcement efforts. Suitable police enforcement efforts included tactics such as directed patrol aggressive disorder enforcement as well as community and problem‐oriented policing explicitly designed to control crime by addressing disorder. Studies that used randomized controlled experimental or quasiexperimental designs were selected. The control group in each study received routine levels of traditional police enforcement tactics.

### 3.5 Data collection and analysis

Twenty‐eight studies containing 30 tests of disorder policing interventions were identified and full narratives of these studies were reported. Nine of the selected studies used randomized experimental designs and 21 used quasiexperimental designs. A formal meta‐analysis was conducted to determine the crime prevention effects of the eligible studies. Random effects models were used to calculate mean effect sizes.

### 3.6 Results

Nineteen of 30 tests of disorder policing interventions reported noteworthy crime reductions. Our meta‐analysis suggests that policing disorder strategies are associated with an overall statistically significant, modest crime reduction effect. The strongest program effect sizes were generated by community and problem‐solving interventions designed to change social and physical disorder conditions at particular places. Conversely, aggressive order maintenance strategies that target disorderly behaviors by individuals in specific areas did not generate significant crime reductions. Crime displacement and diffusion effects were measured in 15 policing disorder tests. Our meta‐analysis suggests disorder policing interventions are associated with diffusion of crime control benefits in areas surrounding targeted locations.

### 3.7 Authors' conclusions

The types of strategies used by police departments to address disorder seem to matter in controlling crime, and this holds important implications for police–community relations, justice, and crime prevention. Further research is needed to understand the key programmatic elements that maximize the capacity of these strategies to prevent crime.

## 4 BACKGROUND

### 4.1 The issue

Dealing with physical and social disorder, or “fixing broken windows,” has become a central element of crime prevention strategies adopted by many American police departments (Kelling & Coles, 1996; Sousa & Kelling, 2006). The general idea of dealing with disorderly conditions to prevent crime is present in myriad police strategies. These range from “order maintenance” and “zero‐tolerance” policing, where the police attempt to impose order through strict enforcement, to “community” and “problem‐oriented policing,” where police attempt to produce order and reduce crime through cooperation with community members and by addressing specific recurring problems (Cordner, 1998; Eck & Maguire, 2006; Skogan, 2006). While its application can vary within and across police departments, disorder policing is now a common crime control strategy.

Most narrative reviews of the crime control effectiveness of policing disorder strategies suggest that the results are mixed (see, e.g., Harcourt & Ludwig, 2006; Kelling & Sousa, 2001). For instance, after reviewing a series of evaluations on the role disorder policing may have played in New York City's crime drop during the 1990s, the National Research Council's Committee to Review Police Policy and Practices concluded that these studies did not provide clear evidence of effectiveness (Skogan & Frydl, 2004). Given the mixed policy evaluation findings, and the popularity of policing disorder, a systematic review of the existing empirical evidence seems warranted. In this review, we synthesize the existing published and unpublished empirical evidence on the effects of disorder policing interventions and provide a systematic assessment of the crime reduction potential of these strategies.

### 4.2 Policing disorder

New York City has been center stage in policy and scholarly debates about policing disorder and the broken windows perspective (most recently, see Rosenfeld, Terry, & Chauhan, 2014; Zimring, 2012). While local officials and national observers attribute the city's violent crime drop in the 1990s to the adoption of a disorder policing strategy, many academics argue that it is difficult to credit this specific strategy with the surprising reduction in violent crime. The New York Police Department (NYPD) implemented the disorder policing strategy within a larger set of organizational changes framed by the Compstat management accountability structure for allocating police resources (Silverman, 1999). As such, it is difficult to disentangle the independent effects of disorder policing relative to other strategies implemented as part of the Compstat process (Weisburd, Mastrofski, McNally, Greenspan, & Willis, 2003). Other scholars suggest that a number of rival causal factors, such as the decline in the city's crack epidemic, played a more important role in the crime drop (Bowling, 1999). Some academics have argued that the crime rate was already declining in the city before the implementation of police reforms, and that the city's decline in homicide rates was not significantly different from declines experienced in surrounding states and in other large cities that did not implement aggressive enforcement policies during that time period (Baumer & Wolff, 2014; Eck & Maguire, 2006).

Since the NYPD implemented its post‐1993 changes as a city‐wide crime control strategy, it was not possible for evaluators to utilize a rigorous evaluation design. However, a series of sophisticated statistical analyses have examined the effects of policing disorder on violent crime trends in New York City. These studies represent very careful attempts to determine whether disorder policing can be associated with the city's crime drop, by controlling statistically for rival causal factors, such as the decline in the city's crack epidemic and relevant sociodemographic, economic, and criminal justice changes over the course of the 1990s. These studies generally can be distinguished by differences in modeling techniques, dependent variables, time series length, extensiveness of control variables included in the analysis, the functional form of control variables, and measurement levels (e.g., precincts vs. boroughs). These studies commonly use increases in misdemeanor arrests, or combined ordinance‐violation and misdemeanor arrests, as the key measures of the NYPD policing disorder strategy.

These nonexperimental analyses have generally found statistically significant associations between the NYPD policing disorder strategy and decreased violent crime, with effects ranging from modest (Cerda et al., 2009; Chauhan et al., 2011; Messner et al., 2007; Rosenfeld, Fornango, & Rengifo, 2007) to large (Corman & Mocan, 2005; Kelling & Sousa, 2001). Harcourt and Ludwig (2006) and Greenberg (2014) report no statistically significant violence reduction impacts associated with the NYPD strategy. While this body of evidence seems to suggest that the NYPD policing disorder strategy may have generated violence reduction impacts, the magnitude of effects remains unclear.

### 4.3 How the intervention might work

In their seminal “broken windows” article, Wilson and Kelling (1982) argue that social incivilities (e.g., loitering, public drinking, and prostitution) and physical incivilities (e.g., vacant lots, trash, and abandoned buildings) cause residents and workers in a neighborhood to be fearful. Fear causes many stable families to move out of the neighborhood and the remaining residents isolate themselves and avoid others. Anonymity increases and the level of informal social control decreases. The lack of control and escalating disorder attracts more potential offenders to the area and this increases serious criminal behavior. Wilson and Kelling (1982) argue that serious crime develops because the police and citizens do not work together to prevent urban decay and social disorder.

Several scholars suggest a strong need to establish a clearer distinction between crime and disorder (e.g., see Gau and Pratt, 2000). This comes up in the context of observational studies as well as evaluation studies. Here, the matter is about trying to avoid confounding measures of disorder with measures of crime. This remains a salient critique of the broken windows perspective. Weisburd et al. (2015), for instance, propose that the focus should be on serious crime and violent crime in particular; less serious crime should not be measured. They also call for a greater focus on physical disorder and more direct measures, including loitering, disorderly conduct, and drinking or intoxication.

The available research evidence on the theoretical connections between disorder and more serious crime is mixed. In the Netherlands, Keizer et al. (2008) conducted six field experiments examining the links between disorder and more serious crime and concluded that dealing with disorderly conditions was an important intervention to halt the spread of further crime and disorder. Skogan's (1990) survey research found disorder to be significantly correlated with perceived crime problems in a neighborhood even after controlling for the population's poverty, stability, and racial composition. Further, Skogan's (1990) analysis of robbery victimization data from 30 neighborhoods found that economic and social factors' links to crime were indirect and mediated through disorder. In his reanalysis of the Skogan data, Harcourt (2001, 2001) removed several neighborhoods with very strong disorder‐crime connections from Newark, New Jersey, and reported no significant relationship between disorder and more serious crime in the remaining neighborhoods. Eck and Maguire (2006) suggest that Harcourt's analyses do not disprove Skogan's results; rather his analyses simply document that the data are sensitive to outliers. Indeed, the removal of different neighborhoods from Harcourt's analysis may have strengthened the disorder‐crime connection (Eck & Maguire, 2006).

In his longitudinal analysis of Baltimore neighborhoods, Taylor (2001) finds some support that disorderly conditions lead to more serious crime. However, these results varied according to types of disorder and types of crime. Taylor (2001) suggests that other indicators, such as initial neighborhood status, are more consistent predictors of later serious crimes. Using systematic social observation data to capture social and physical incivilities on the streets of Chicago, Sampson and Raudenbush (1999) found that, with the exception of robbery, public disorder was not significantly related to most forms of serious crime when neighborhood characteristics such as poverty, stability, race, and collective efficacy were considered. Sampson and Raudenbush's findings have been criticized because their social observation data on disorder were collected during the day rather than at night (Sousa & Kelling, 2006), as well as based on their decision to test a model in which disorder mediates the effects of neighborhood characteristics on crime rather than neighborhood characteristics mediating the effects of disorder on crime (Jang & Johnson, 2001). In another analysis, Xu et al. (2005) point out that Sampson and Raudenbush's (1999) results actually are supportive of broken windows theory.

Research on high activity crime places reveals that disorder clusters in space and time with more serious crimes. In their closer look at crime in Minneapolis hot spots, Weisburd et al. (1992) found that assault calls for service and robbery of person calls for service were significantly correlated with “drunken person” calls for service. In Jersey City, New Jersey, Braga et al. (1999) found that high‐activity violent crime places also suffered from serious disorder problems. The concentration of disorder in small places provides compelling opportunities for criminals. As Braga (2008) describes, abandoned buildings and vacant lots provide unguarded places for drug dealers selling their product and concealment for robbers looking to ambush an unsuspecting passerby. Disorder policing strategies that modify the crime opportunity structure at specific places by addressing social incivilities and physical incivilities could have important impacts on criminal behavior.

### 4.4 Why it is important to do the review

The scientific research evidence on the crime control effectiveness of broad‐based disorder policing strategies, such as quality‐of‐life programs and order maintenance enforcement practices, is mixed. While nonexperimental evidence seems to suggest that the NYPD policing disorder strategy may have generated violence reduction impacts, the magnitude of effects remains unclear. More rigorous policy evaluations implemented in other jurisdictions support the perspective that dealing with disorderly conditions generates crime control gains. Two separate randomized controlled trials of disorder policing strategies implemented within a problem‐oriented policing framework found the strategy resulted in significant reductions in calls for service to the police in Jersey City, New Jersey (Braga et al., 1999), and Lowell, Massachusetts (Braga & Bond, 2008).

A quasiexperimental evaluation of the Safer City Initiative, an intervention launched by the Los Angeles Police Department to reduce homeless‐related crimes by addressing disorderly conditions associated with homeless encampments, generated modest reductions in violent, property, and nuisance street crimes (Berk & MacDonald, 2010). Other macro‐level analyses have generated results supportive of broad‐based policing disorder strategies. In California, controlling for demographic, economic, and deterrence variables, a county‐level analysis revealed that increases in misdemeanor arrests was associated with significant decreases in felony property offenses (Worrall, 2002). Finally, an analysis of robbery rates in 156 American cities revealed that aggressive policing of disorderly conduct and driving under the influence reduces robbery (Sampson & Cohen, 1988).

Other evaluations have not found significant crime prevention gains associated with broad‐based policing disorder strategies. A recent reanalysis of the Kelling and Sousa (2001) data did not find that a generalized broken windows strategy, as measured by increased misdemeanor arrests, yielded significant reductions in serious crimes in New York City between 1989 and 1998 (Harcourt & Ludwig, 2006). An evaluation of a quality‐of‐life policing initiative focused on social and physical disorder in four target zones in Chandler, Arizona, did not find any significant reductions in serious crime associated with the strategy (Katz, Webb, & Schaefer, 2001). An evaluation of a 1‐month police enforcement effort to reduce alcohol and traffic‐related offenses in a community in a Midwestern city did not find any significant reductions in robbery or burglary in the targeted area (Novak, Hartman, Holsinger, & Turner, 1999). Similarly, a randomized controlled experiment of broken windows policing in three towns in California (Redlands, Colton, and Ontario) found no significant effects on fear of crime, police legitimacy, collective efficacy, or perceptions of crime and social disorder (Weisburd et al., 2012).

Disorder policing strategies are common crime prevention interventions implemented by police departments across the world. Given the ubiquity of disorder policing and the mixed program evaluation findings presented here, a systematic review of the existing empirical evidence is warranted.

## 5 OBJECTIVES

This review synthesizes the existing published and nonpublished empirical evidence on the effects of disorder policing interventions and provides a systematic assessment of the crime reduction value of disorder policing in neighborhoods. The review also examines whether policing disorder strategies cause crime displacement or diffusion of crime control benefits in areas immediately surrounding targeted locations. It is anticipated that this review will help inform policy makers and police department decision makers regarding the continued use of disorder policing interventions to reduce crime in neighborhoods. Many police agencies in the United States, United Kingdom, Australia, and other nations currently use disorder policing as a core crime control strategy, and a critical examination of the existing evidence is warranted.

## 6 METHODS

This review synthesizes the existing published and nonpublished empirical evidence on the effects of disorder policing interventions and provides a systematic assessment of the crime reduction value of disorder policing in neighborhoods. In keeping with the conventions established by the Campbell Collaboration, the stages of this systematic review and the criteria used to select eligible studies are described below.

### 6.1 Criteria for considering studies for this review

#### 6.1.1 Types of studies

Studies that use comparison group designs, such as randomized controlled trials and quasiexperimental designs (Shadish, Cook, & Campbell, 2002), were eligible for the main analyses of this review. Only the most rigorous quasiexperimental designs were included, with the minimum design involving before and after measures of crime in treatment and control areas. In many controlled policing disorder evaluations (e.g., Berk & MacDonald, 2010), the control group experiences routine modern police responses to crime. Control areas usually experience a blend of traditional police responses (e.g., random patrol, rapid response, and ad‐hoc investigations) and opportunistic community problem‐solving responses. While disorder interventions developed from community policing initiatives may be present in certain control areas, none of the control areas can engage disorder policing strategies as their main approach to address crime problems.

#### 6.1.2 Types of areas

Only area‐level studies were included in our systematic review. Eligible areas can range from small places (such as hot spots comprised of clusters of street segments or addresses) to police defined areas (such as districts, precincts, sectors, or beats) to larger neighborhood units (such as census tracts or a researcher‐defined area). On the basis of the the selected literature review, we expected that our research strategy would yield a diverse set of targeted areas across the identified policing disorder studies. For example, evaluations of disorder policing strategies in New York City analyzed the city‐wide effects of the strategy at different units of analysis such as police precincts and police boroughs (Corman & Mocan, 2005; Harcourt & Ludwig, 2006; Kelling & Sousa, 2001; Messner et al., 2007; Rosenfeld et al., 2007). In Los Angeles, evaluators assessed the impact of a policing disorder strategy by comparing crime trends in one treatment police division area relative to crime trends in four adjacent comparison police division areas (Berk & MacDonald, 2010). In the Jersey City and Lowell randomized controlled trials, the units of analysis were crime “hot spots” comprising street block faces and street intersections (Braga et al., 1999; Braga & Bond, 2008).

It is important to note that this heterogeneity in the units of analysis across studies could have varying and policy‐relevant effects on crime prevention outcomes associated with the policing disorder strategies. As such, we classified the types of areas to ensure that the review is measuring similar findings across the potentially diverse set of locations subjected to treatment. We distinguished between small areas such as hot spots and buildings, smaller police‐defined units (such as beats), larger police‐defined units (such as districts and precincts), larger areas (such as neighborhoods and communities), and other spatial units.

#### 6.1.3 Types of interventions

The general idea of dealing with disorderly conditions to prevent crime is present in myriad police strategies, ranging from “order maintenance” and “zero‐tolerance,” where the police attempt to impose order through strict enforcement, to “community” and “problem‐oriented policing” strategies, where police attempt to produce order and reduce crime through cooperation with community members and by addressing specific recurring problems (Cordner, 1998; Eck & Maguire, 2006; Skogan, 2006; Skogan et al., 1999). Problem‐oriented policing programs that did not attempt to control crime by reducing disorder were excluded from this review.

We considered all policing programs that attempt to reduce crime through addressing physical disorder (vacant lots, abandoned buildings, graffiti, etc.) and social disorder (public drinking, prostitution, loitering, etc.) in neighborhood areas. These interventions were compared to other police crime reduction efforts that do not attempt to reduce crime through reducing disorderly conditions, such as traditional policing (i.e., regular levels of patrol, ad‐hoc investigations, etc.).

#### 6.1.4 Types of outcome measures

Eligible studies measured the effects of the disorder policing intervention on officially recorded levels of crime in areas such as crime incident reports, citizen emergency calls for service, and arrest data. Other outcome measures such as survey, interview, systematic observations of social disorder (such as loitering, public drinking, and the solicitation of prostitution), systematic observations of physical disorder (such as trash, broken windows, graffiti, abandoned homes, and vacant lots), and victimization measures used by eligible studies to measure program effectiveness were coded and analyzed.

Since area‐level studies were included in this review, particular attention was paid to studies that measured spatial crime displacement effects and diffusion of crime control effects. Policing strategies focused on specific locations have been criticized as resulting in displacement (see Reppetto, 1976). Academics have observed that crime prevention programs may result in the complete opposite of displacement—that crime control benefits were greater than expected and “spill over” into places beyond the target areas (Clarke & Weisburd, 1994). The quality of the methodologies used to measure displacement and diffusion effects, as well as the types of displacement examined (spatial, temporal, target, modus operandi), were assessed.

### 6.2 Search methods for identification of studies

To identify the studies meeting the criteria of this review, several search strategies were used. First, a keyword search was performed on an array of online abstract databases (see lists of keywords and databases below). Second, the bibliographies of past narrative and empirical reviews of literature that examined the effectiveness of police crime control programs were reviewed (e.g., Braga, 2008; Eck & Maguire, 2006; Sherman, 1997, 2002; Skogan & Frydl, 2004; Weisburd & Eck, 2004). Third, forward searches for works that cited seminal disorder policing studies were performed (e.g., Kelling & Coles, 1996; Kelling & Sousa, 2001; Wilson & Kelling, 1982). Fourth, bibliographies of past completed Campbell systematic reviews of police crime control efforts were searched (Bowers, Johnson, Guerette, Summers, & Poynton, 2011; Braga, 2007; Braga, Papachristos, & Hureau, 2012; Braga & Weisburd, 2012; Mazerolle, Soole, & Rombouts, 2007; Weisburd, Telep, Hinkle, & Eck, 2008).

Fifth, hand searches of leading journals in the field were performed. All of the searches were completed up to the end of 2012. Thus, the review covers studies published in 2012 and earlier. Sixth, searches were carried out of policing literature available on governmental and nonprofit organization web pages. Seventh, keyword searches of grey literature databases (e.g., Gottfredson Library criminal justice grey literature database at Rutgers School of Criminal Justice maintained by Phyllis Schultze; the System for Grey Literature in Europe, http://www.opengrey.eu/) were performed. Eighth, keyword searches of abstracts of papers presented at professional criminology and criminal justice conferences (e.g. American Society of Criminology, Academy of Criminal Justice Sciences, International Society of Criminology, U.S. National Institute of Justice Research and Evaluation Conference) were conducted. Ninth, after finishing the above searches and reviewing the studies as described later, the list of studies meeting our eligibility criteria was emailed (in July 2013) to leading criminology and criminal justice scholars knowledgeable in the area of disorder policing strategies. These 120 scholars were defined as those who authored at least one study that appeared on our inclusion list, anyone involved with the National Academy of Sciences review of police research (Skogan & Frydl, 2004), and other leading scholars (see Appendix 1). This helped to identify any studies overlooked by the above searches—as these experts may be able to make referrals to studies that were missed, particularly unpublished studies.

Finally, an information specialist was engaged at the outset of our review and at points along the way to ensure that appropriate search strategies were used to identify the studies meeting the criteria of this review. For instance, we worked with the information specialist to conduct an extensive Google search for eligible studies by using the search terms below, as well as including words such as “research,” “evaluation,” and “program analysis.” The information specialist was consulted on the use of Google Scholar to identify studies that cite seminal policing disorder studies (e.g., Kelling & Coles, 1996; Wilson & Kelling, 1982) and on the use of publisher databases and indexes (e.g., Wiley, Sage, and Springer) to identify potentially eligible studies.

The following 15 databases were searched:

1. Criminal Justice Periodical Index
2. Sociological Abstracts
3. Social Science Abstracts (SocialSciAbs)
4. Social Science Citation Index
5. Arts and Humanities Search (AHSearch)
6. Criminal Justice Abstracts
7. National Criminal Justice Reference Service (NCJRS) Abstracts
8. Educational Resources Information Clearinghouse (ERIC)
9. Legal Resource Index
10. Dissertation Abstracts
11. Government Publications Office, Monthly Catalog (GPO Monthly)
12. Google Scholar
13. Online Computer Library Center (OCLC) SearchFirst
14. CINCH data search
15. Academic Search Premier

The following terms were used to search the above databases:

1. Broken windows AND police
2. Disorder AND police
3. Incivilities AND police
4. Disorder policing
5. Order maintenance policing
6. Zero tolerance policing
7. Quality of life policing
8. Misdemeanor arrest policing
9. Signal crimes

Terms listed in items 1–9 were also connected via the use of Boolean “or” terms during the search. In addition, two existing registers of randomized controlled trials were consulted. These include (a) the “Registry of Experiments in Criminal Sanctions, 1950–1983 (Weisburd, Sherman, & Petrosino, 1990) and (b) the “Social, Psychological, Educational, and Criminological Trials Register” or C2 SPECTR being developed by the United Kingdom Cochrane Centre and the University of Pennsylvania (Turner et al., 2003). Two additional online databases of rigorous studies in policing were reviewed: Evidence‐Based Policing Matrix (http://www.policingmatrix.org) and the U.S. Office of Justice Program's CrimeSolutions.gov website.

### 6.3 Data collection and analysis

#### 6.3.1 Details of study coding categories

All eligible studies were coded (see coding protocol; Appendix 2) on a variety of criteria including:

a. Reference information (title, authors, publication, etc.)
b. Nature of description of selection of site, problems, and so forth.
c. Nature and description of selection of comparison group or period
d. Unit of analysis
e. Sample size
f. Methodological type (randomized experiment or quasiexperiment)
g. Description of the disorder policing intervention
h. Dosage intensity and type
i. Implementation difficulties
j. Statistical test(s) used
k. Reports of statistical significance (if any)
l. Effect size/power (if any)
m. Conclusions drawn by the authors

Two of the three authors (Schnell, Braga) independently coded each eligible study. Where there were discrepancies, all three authors jointly reviewed the study and determined the final coding decision.

#### 6.3.2 Statistical procedures and conventions

Analysis of outcome measures across studies were carried out in a uniform manner and, when appropriate and possible, involved quantitative analytical methods. We used meta‐analyses of program effects to determine the size and direction of the effects and to weight effect sizes based on the variance of the effect size and the study sample size (Lipsey & Wilson, 2001). In this systematic review, the standardized mean difference effect size (also known as Cohen's *d*; see Rosenthal, 1994) was used. We were able to calculate effect sizes for 30 main effects tests in the 28 eligible studies. Computation of effect sizes in the studies was not always direct. The goal was to convert all observed effects into a standardized mean difference effect size metric. Indeed, it was sometimes difficult to develop precise effect size metrics from published materials. This reflects a more general problem in crime and justice with “reporting validity” (Farrington, 2003; Lösel & Köferl, 1989), and has been documented in reviews of reporting validity in crime and justice studies (see Perry & Johnson, 2008; Perry, Weisburd, & Hewitt, 2010).

The Effect Size Calculator, developed by David B. Wilson and available on the Campbell Collaboration's website, was used to calculate standardized mean difference effect sizes for reported outcomes in each study.^1^ Biostat's Comprehensive Meta Analysis Version 2.2 was then used to conduct the meta‐analysis of effect sizes. For many of the included studies, treatment and control group crime counts were used to calculate effect sizes. From these raw counts, Odds ratios (ORs) were first calculated. To obtain Cohen's *d*, the log of this OR was then multiplied by √3/π (Hasselblad & Hedges, 1995). The variance of log OR was calculated as the sum of the reciprocal terms in the cells immediately below. The computational formulae are presented here:

$$\begin{array}{ccc} & \text{Pr}e & Post\\ Treatment & a & b\\ Control & c & d \end{array}$$

OR = (*b* × c)/(*a* × *d*)

V(LOR) = (1/*a*) + (1/*b*) + (1/*c*) + (1/*d*)

An adjustment for over‐dispersion was then made using the method in Farrington et al. (2007): the adjusted V(LOR) is computed as the product of V(LOR) and *D*, with *D* = 0.0008 ×*N* + 1.2. *N* is indexed as the mean number of incidents per case and is calculated as the total number of incidents (*a* + *b* + *c* + *d*) divided by the total number of treatment plus control cases. This adjusted V(LOR) is then multiplied by (3/π^2^) to give the final variance of the effect size [*V*(*d*)] (Hasselblad & Hedges, 1995).

In other included studies, Cohen's *d* could not be estimated in the way described above, and other methods were pursued. For instance, in the Jersey City Drug Market Analysis Program (DMAP) experiment and the Los Angeles Suburban Broken Windows Project, the *p* levels from a mixed‐model analysis of variance were used to compute the effect sizes. The *p* level for each contrast was first converted to a Z score which was then used to calculate a correlational effect size (*r*). Using conventional formulae, this effect size was then converted to Cohen's *d*. For the Newark Signs of Crime Program, Newark Community Policing Program, Dayton Traffic Enforcement Project, Midwestern City Disorder Project, Jersey City Displacement and Diffusion Study, and Los Angeles Safer Cities Initiative, we calculated standardized mean effect sizes based on the *t* test results reported for the intervention variables' effects on the outcome variables.

#### 6.3.3 Determination of independent findings

One problem in conducting meta‐analyses in crime and justice is that investigators often do not prioritize outcomes examined. This is common in studies in the social sciences in which authors consider it good practice to report all relevant outcomes. For example, the Jersey City DMAP experiment presents an array of outcome measures, including violence, property, disorder, and narcotics calls for service (Weisburd & Green, 1995). However, the lack of prioritization of outcomes in a study raises the question of how to derive an overall effect of treatment. Specifically, the reporting of one significant result may reflect a type of “creaming” in which the authors focus on one significant finding while ignoring the less positive results of other outcomes. But authors commonly view the presentation of multiple findings as a method for identifying the specific contexts in which the treatment is effective. When the number of such comparisons is small and therefore unlikely to affect the error rates for specific comparisons, such an approach is often valid.

All studies for which a standardized effect size could be obtained were analyzed using three approaches and served as a sensitivity analysis to evaluate the effects of input variation on the output variation. The first approach is conservative; we calculated an overall mean effect size for each study that combined all reported outcomes. The second represents the largest effect reported in the studies and offers an upper bound to the review findings. It is important to note that in some of the studies with more than one outcome reported, the largest outcome reflected what authors thought would be the most direct program effect. This was true for the Jersey City DMAP experiment, which examined a wider range of crime outcome measures, but suggested that the largest program effects would be found in the case of disorder calls of service given the program's focus on street‐level drug markets (Weisburd & Green, 1995). Finally, the smallest effect size for each study was analyzed. This approach is the most conservative and likely underestimates the effect of disorder policing programs on crime. It was used here primarily to provide a lower bound to the review findings.

#### 6.3.4 Treatment of qualitative research

Qualitative research on crime and disorder outcomes was not included in this systematic review. The authors hope that a qualitative researcher will assist in future updates to this review with a synthesis of qualitative evaluation measures.

## 7 RESULTS

### 7.1 Description of studies

#### 7.1.1 Results of the search

Search strategies in the systematic review process generate a large number of citations and abstracts for potentially relevant studies that must be closely screened to determine whether the studies meet the eligibility criteria (Farrington & Petrosino, 2001). The screening process yields a much smaller pool of eligible studies for inclusion in the review. The search strategies produced 8,420 distinct abstracts using the nine keywords and 15 databases. The contents of the 8,420 abstracts were reviewed for any suggestion of an experimental or quasiexperimental evaluation of disorder policing interventions. Two hundred sixty‐nine distinct abstracts were selected for closer review and the full‐text reports, journal articles, and books for these abstracts were acquired and carefully assessed to determine whether the interventions involved disorder policing interventions and whether the studies used randomized controlled trial designs or nonrandomized quasiexperimental designs. Altogether, 28 eligible studies containing 30 independent tests of policing disorder interventions were identified and included in this review:

1. Newark Signs of Crime Program (Pate & Skogan, 1985a)
2. Newark Community Policing Program (Pate & Skogan, 1985b)
3. Southeastern City Foot Patrol Project (Esbensen, 1987)
4. New York Community Patrol Officer Program (McElroy, Cosgrove, & Sadd, 1990)
5. Jersey City Drug Market Analysis Program (Weisburd & Green, 1995)
6. Dayton Traffic Enforcement Project (Weiss & Freels, 1996)
7. San Diego Slumlords Project (Clarke & Bichler‐Robertson, 1998)
8. San Diego Place Managers Project (Eck & Wartell, 1998)
9. Midwestern City Disorder Project (Novak et al., 1999)
10. Jersey City Problem‐Oriented Policing Project (Braga et al., 1999)
11. Spokane Public Housing Project (McGarrell, Giacomazzi, & Thurman, 1999)
12. Oakland Beat Health Program (Mazerolle, Price, & Roehl, 2000)
13. Chicago Nuisance Abatement Program (Higgins & Coldren, 2000)
14. Richmond Weed and Seed Initiative (Smith, 2001)
15. Wales Zero Tolerance Initiative (Rogers, 2002)
16. Detroit Antigang Initiative (Bynum & Varano, 2003)
17. St. Louis Antigang Initiative (Decker & Curry, 2003)
18. Jersey City Displacement Study (Weisburd et al., 2006)
19. Los Angeles Baldwin Safer Cities Initiative (Wagers, 2007)
20. Lowell Problem‐Oriented Policing Project (Braga & Bond, 2008)
21. New Britain Weed and Seed Project (Costanza, Helms, Ratansi, Kilburn, & Harmon, 2010)
22. Las Vegas Order Maintenance Project (Pace, 2010)
23. Los Angeles Safer Cities Initiative (Berk & MacDonald, 2010)
24. Boston Smart Policing Initiative (Braga, Hureau, & Papachristos, 2011)
25. Jacksonville Hot Spots Policing Project (Taylor, Koper, & Woods, 2011)
26. London Safe as Houses Project (Enfield Police Department, 2011)
27. Los Angeles Suburbs Broken Windows Project (Weisburd, Hinkle, Famega, & Ready, 2012)
28. Lowell Smart Policing Initiative (Bond & Hajjar, 2013)

The Jersey City Displacement Study involved two separate tests of policing disorder interventions to control one prostitution hot spot and one drug crime hot spot (Weisburd et al., 2006). The Detroit Antigang Initiative involved distinct tests of policing disorder interventions implemented in the two separate policing districts (Bynum & Varano, 2003).

#### 7.1.2 Characteristics of selected studies

Table 1 presents the basic characteristics of the 30 eligible tests. Twenty‐eight of the 30 tests (93.3%) were conducted in the United States, with the remaining two conducted in the United Kingdom. Twelve tests (40.0%) were completed in large cities with more than 500,000 residents, nine tests (30.0%) were completed in medium‐sized cities with 200,000–500,000 residents, and nine tests (30.0%) were completed in smaller cities with <200,000 residents. Six cities were the research sites for multiple policing disorder evaluations. Jersey City (NJ) was the site for four tests, while Detroit (MI), Los Angeles (CA), Lowell (MA), Newark (NJ), and San Diego (CA) were the sites for two tests each. Seventeen of the eligible policing disorder tests were published in peer‐reviewed journals (56.7%), four were published as chapters in edited books (13.3%), one was available as a published report (3.3%), and eight were available as unpublished reports, including doctoral dissertations and masters' theses (26.7%). Twenty‐one tests used quasiexperimental designs (70.0%) and nine used randomized experimental designs (30.0%).

**Table 1:** Key characteristics of eligible disorder policing studies (*N *= 30)

Characteristics	*N*	Percent
Evaluation country		
United States	28	93.3
United Kingdom	2	6.7
City population		
Small (<200,000 residents)	9	30.0
Medium (200,000–500,000 residents)	9	30.0
Large (>500,000 residents)	12	40.0
Evaluation type		
Randomized controlled trial	9	30.0
Quasiexperimental design	21	70.0
Publication type		
Peer‐reviewed journal	17	56.7
Unpublished technical report, dissertation/thesis	8	26.7
Edited book chapter	4	13.3
Published technical report	1	3.3
Unit of analysis		
Small places (e.g., crime hot spots and buildings)	14	46.7
Smaller police‐defined units (e.g., beats)	8	26.7
Neighborhoods/highway segments	4	13.3
Larger police‐defined units (e.g., precincts, divisions)	4	13.3
Intervention strategy		
Community problem‐solving strategy/place	21	70.0
Aggressive order maintenance/people	9	30.0

Units of analysis included small places (such as crime hot spots and problem buildings; 46.7%), smaller police‐defined areas (such as beats; 26.7%), neighborhoods and selected stretches of highways (13.3%), and larger police‐defined areas (such as precincts and divisions; 13.3%). We also found a diversity of strategies and tactics used by police departments in these programs to address social and physical disorder problems. Our review suggested that there are two main types of policing disorder interventions: (a) increased use of aggressive order maintenance techniques to reduce disorderly behavior by individuals, and (b) community problem‐solving approaches that seek to change social and physical disorder conditions at particular places.

Table 2 provides a brief summary of the eligible studies as organized by the two main types of disorder policing. The community problem‐solving programs, which accounted for 20 of the 30 tests, usually attempted to engage residents, local merchants, and others in the identification of local crime and disorder problems and the development and implementation of appropriate responses. As such, the community problem‐solving programs often involved a varied set of disorder reduction strategies designed to change criminogenic dynamics generated by social and physical disorder problems in highly specific places. The aggressive order maintenance strategies, which accounted for the other 10 tests, primarily used arrests, ordinance violation summons, and other law enforcement strategies to target disorderly individuals, usually in larger areas. While community members were sometimes engaged in the process, they were generally not involved in the aggressive order maintenance programs in any substantive way.

**Table 2:** Disorder policing programs by type

Study	Intervention
*Community problem‐solving to address social and physical disorder at places*	
Boston Smart Policing Initiative	Problem‐oriented policing strategy that largely targeted social and physical disorder in violent crime hot spots
Braga et al. (2011)	
Chicago Nuisance Abatement	Police engaged building owners and managers to address gangs and illegal drug selling by dealing with physical and social disorder
Higgins and Coldren (2000)	
Jacksonville Hot Spots Policing	Problem‐oriented policing strategy that largely targeted social and physical disorder in violent crime hot spots
Taylor et al. (2011)	
Jersey City Drug Market Analysis	Problem‐oriented policing strategy to control drug markets by arresting drug sellers and changing disorderly conditions
Weisburd and Green (1995)	
Jersey City Problem‐Oriented Policing	Problem‐oriented policing strategy that largely targeted social and physical disorder in violent crime hot spots
Braga et al. (1999)	
Jersey City Displacement Study	Problem‐oriented policing used to target high‐rate offenders and disorderly conditions in drug and prostitution hot spots (two independent tests)
Weisburd et al. (2006)	
London Safe as Houses	Situational prevention measures and improvements to disorderly physical conditions to reduce repeat burglaries
Enfield Police Department (2011)	
Los Angeles Safer Cities Initiative	Place‐based policing intervention to eliminate social and physical disorder created by homeless encampments
Berk and MacDonald (2010)	
Los Angeles SCI Baldwin	Broken windows policing strategy, guided by community problem‐solving concepts, to control crime in three areas
Wagers (2007)	
LA Suburbs Broken Windows	Broken windows policing strategy that targeted social and physical disorder at high‐crime street segments in three cities
Weisburd et al. (2012)	
Lowell Smart Policing Initiative	Community problem‐solving intervention to address social and physical disorder in property crime hot spots
Bond and Hajjar (2013)	
Lowell Problem‐Oriented Policing	Problem‐oriented policing strategy that largely targeted social and physical disorder in crime hot spots
Braga and Bond (2008)	
New York Community Patrol Officer	Community problem‐solving officers that mostly addressed social and physical disorder problems in their beats
McElroy et al. (1990)	
Newark Signs of Crime	Police collaborated with public and private agencies to reduce fear and crime by addressing social and physical disorder
Pate and Skogan (1985a)	
Newark Community Policing	Coordinated community policing program to reduce fear and crime by addressing social and physical disorder
Pate and Skogan (1985b)	
Oakland Beat Health	Property owners and building managers required to address illicit drug sales by dealing with physical and social disorder
Mazerolle et al. (2000)	
San Diego Slumlords	Slumlord forced to use competent building managers to clean physical disorder that facilitated illegal drug dealing
Clarke and Bichler‐Robertson (1998)	
San Diego Place Managers	Property owners and building managers required to address illegal drug sales by dealing with physical and social disorder
Eck and Wartell (1998)	
Spokane Public Housing	Community problem‐solving effort that addressed social and physical disorder problems in public housing facilities
McGarrell et al. (1999)	
*Aggressive order maintenance targeting individual disorderly behaviors*	
Dayton Traffic Enforcement	Aggressive enforcement of traffic laws to reduce more serious crimes on highway segments
Weiss and Freels (1996)	
Detroit Anti‐Gang Initiative	Traditional suppression and aggressive order maintenance actions targeting gang members in 4th and 9th Precincts (two independent tests)
Bynum and Varano (2003)	
New Britain Weed and Seed	Community policing program that was more focused on arresting offenders than disorder problems in targeted zone
Costanza et al. (2010)	
St. Louis Anti‐Gang Initiative	Traditional suppression and aggressive order maintenance actions targeting gang members in 5th District
Decker and Curry (2003)	
Southeastern City Foot Patrol	Vagrants, prostitutes, drunkards, and parking violators targeted by foot patrol officers in downtown business area
Esbensen (1987)	
Midwestern City Disorder	Aggressive enforcement crackdown on public drinking, speeding, and other social disorders in 10 by 12 block area
Novak et al. (1999)	
Las Vegas Order Maintenance	Specialized unit dedicated to maintaining order and enforcing misdemeanor arrests laws in targeted area
Pace (2010)	
Wales Zero Tolerance	Zero tolerance community safety strategy to improve quality of life by targeting vandalism and youth disorder
Rogers (2002)	
Richmond Weed and Seed	Community policing program that was more focused on arresting offenders than disorder problems in targeted zone
Smith (2001)	

Community problem‐solving programs used two distinct methods to target physical and social disorder. The first method emphasizes the development of larger problem‐solving efforts—such as collaborative working groups formed with community stakeholders—to collectively identify creative responses to address physical and social disorder problems. This set of responses is then applied uniformly across all units of analysis, which in turn provides a singular, composite treatment to disorder in communities. Eight of the 20 community problem‐solving programs used this method. The Newark Community Policing Program (Pate & Skogan, 1985b) used foot patrol, community storefront offices, and home visits to establish a problem‐solving partnership with communities receiving treatment. The Spokane Public Housing Project (McGarrell et al., 1999) and Chicago Nuisance Abatement Program (Higgins & Coldren, 2000) focused on problem housing facilities and formed partnerships with local organizations that could help address disorder at these locations. The London Safe as Houses Project (Enfield Police Department, 2011) collaborated with residents in burglary hot spots to clean‐up physical disorder and provide target‐hardening services to alter the criminogenic conditions of houses. The San Diego Slumlords Project (Clarke & Bichler‐Robertson, 1998) used a unique treatment that relied entirely upon leveraging place managers of apartment complexes to become problem‐solvers to devise solutions to disorder problems in their facilities.

The second method provides training to officers in community problem‐solving techniques and then allows these officers to devise solutions based on the specific criminogenic conditions of the locations they patrol. Eleven of 20 community problem‐solving programs used this method. The New York CPOP (McElroy et al. 1990) trained officers as “planner, problem solver, community organizer, and information exchange link” for specialized neighborhood units (see pp. 7–8). The Jersey City Problem‐Oriented Policing Project (Braga et al., 1999), Lowell Problem‐Oriented Policing Project (Braga & Bond, 2008), and Lowell Smart Policing Initiative (Bond & Hajjar, 2013) used the SARA model in violent crime hot spots as a community problem‐solving strategy. The Oakland Beat Health Program (Mazerolle et al., 2000) offered officers discretion between two choices: to mediate with place managers to remedy disorder problems or target enforcement of other municipal regulatory agencies on locations. The Boston Smart Policing Initiative (Braga et al. 2011) specifically used over 30 different community‐problem solving techniques at crime hot spots, including situational/environmental interventions, enforcement interventions, and community outreach/social service interventions to address both physical and social disorder problems.

Seven of nine aggressive order maintenance programs attempted to use community problem‐solving strategies in their implementation of disorder policing, but the adoption of these techniques was incomplete. The Richmond Weed and Seed Initiative (Smith, 2001) and New Britain Weed and Seed Project (Costanza et al., 2010) proposed the use of intensive enforcement and crackdowns on social disorder (i.e., “weed”) followed by community problem‐solving techniques to reduce crime (i.e., “seed”). The process and outcome evaluations of these interventions just focused on the aggressive order maintenance components of these strategies. The Southeastern City Foot Patrol Project (Esbensen, 1987) emphasized the community problem‐solving technique of foot patrol, while the Wales Zero Tolerance Initiative (Rogers, 2002), Detroit Antigang Initiative (Bynum & Varano, 2003), and Midwestern City Disorder Project (Novak et al., 1999) constructed community partnerships to target disorder—yet these techniques were overshadowed by the programs' use of increased enforcement of social disorder. The Las Vegas Order Maintenance Project (Pace, 2010) even explicitly designed the intervention to follow Wilson and Kelling's (1982) original broken windows thesis, which emphasized community problem‐solving strategies; however, the implementation of the intervention was much closer to an aggressive order maintenance strategy. The Dayton Traffic Enforcement Project (Weiss & Freels, 1996) and the St. Louis Antigang Initiative (Decker & Curry, 2003) were the only two aggressive order maintenance programs that did not even discuss implementation of community‐problem solving techniques.

Both the community problem‐solving and aggressive order maintenance programs deployed similar techniques to reduce physical and social disorder. Aggressive order maintenance programs were primarily concerned with targeting social disorder. Disorder policing strategies used several techniques to reduce social disorder, including increasing the number of arrests and citations of individuals in problem areas for minor offenses (Costanza et al., 2010; Decker & Curry, 2003; Esbensen, 1987; Pace, 2010; Smith, 2001; Weiss & Freels, 1996), applying different enforcement actions to problem locations (Bond and Hajjar, 2013; Bynum & Varano, 2003; Eck & Wartell, 1998), altering physical environments to discourage social disorder (Braga et al., 1999; Enfield Police Department, 2011; Taylor et al., 2011; Weisburd et al., 2006), collaborating with other regulatory agencies to enforce social ordinances (Novak et al., 1999; Pate & Skogan, 1985a; Rogers, 2002), or using statutes to leverage business owners or place managers to discourage social disorder outside of locations (Braga et al., 2011; Weisburd & Green, 1995).

The enforcement of social disorder was often administered as a “crackdown” or intensive enforcement across primarily aggressive order maintenance programs. The techniques used to target physical disorder included having officers removing trash or graffiti (Braga & Bond, 2008; Wagers, 2007; Weisburd et al., 2012), city employees and community residents removing trash or graffiti (Berk & MacDonald, 2010; McGarrell et al., 1999), use of statutes to leverage business owners or place managers to clean‐up problem locations (Clarke & Bichler‐Robertson, 1998; Higgins & Coldren, 2000; Mazerolle et al., 2000), or individuals under criminal justice supervision cleaning up areas as a condition of their sentencing (Pate & Skogan, 1985b).

#### 7.1.3 Descriptive account of reported crime control impacts

This section provides a brief descriptive account of the effects of the included disorder policing interventions on crime as reported by the study authors. Table 3 summarizes the treatments, units of analysis, and research designs. Table 4 summarizes the main effects of the interventions on crime and disorder measures, treatment effects as measured by other nonofficial data sources, and, if measured, the immediate spatial displacement and diffusion of crime control effects.

**Table 3:** Disorder policing randomized experiments and quasiexperiments

Study	Treatment	Unit of analysis	Research design
Newark (NJ) Signs of Crime Program Pate and Skogan (1985a)	Police collaborated with public and private agencies to reduce fear and crime by addressing social and physical disorder 10‐month intervention period. A process evaluation was provided but no major threats to the integrity of the treatment were noted.	Two neighborhoods—approximately 20 sq. blocks each; selected by police department based on preexisting level of disorder and U.S. Census data on community structural characteristic.	Quasiexperiment; one neighborhood as treatment and one neighborhood as control. Interrupted times series analysis of monthly incident counts compared between treatment and control sites.
Newark (NJ) Community Policing Program Pate and Skogan (1985b)	Coordinated community policing program to reduce fear and crime by addressing social and physical disorder 10‐month intervention period. A process evaluation was provided but no major threats to the integrity of the treatment were noted.	Two neighborhoods—approximately 20 sq. blocks each; selected by police department based on preexisting level of disorder and U.S. Census data on community structural characteristic.	Quasiexperiment; one neighborhood as treatment and one neighborhood as control. Interrupted times series analysis of monthly incident counts compared between treatment and control sites.
Southeastern City Foot Patrol Project Esbensen (1987)	Vagrants, prostitutes, drunkards, and parking violators targeted by foot patrol officers in downtown business area. 2‐year intervention period. No threats to the integrity of the treatment reported	Two business districts; treatment area was approximately 5 blocks—no details given on control areas size. Treatment area was selected because of high level of disorder and concentration of businesses within the area.	Quasiexperiment; one treatment district was matched with one control district. Incident counts for four separate months (January, April, July, and October) were compared to pre‐ and posttest measures.
New York (NY) Community Patrol Officer Program (CPOP) McElroy et al. (1990)	Community problem‐solving officers that mostly addressed social and physical disorder problems in their beats. 2‐year intervention period. Detailed analysis provided on the implementation of the intervention. Officers did a better job with the problem solving component compared to community involvement	74 Precincts; identified based on levels of calls for service and heterogeneity in community structural characteristics from four of the five boroughs in New York City, excluding Staten Island. Also, these precincts were selected because they already had a functioning community policing unit.	Quasiexperiment; 37 CPOP precincts were matched with 37 non‐CPOP precincts. Differences in difference of annual mean counts for incident reports and calls for service were compared between years for treatment and control.
Jersey City (NJ) Drug Market Analysis Program (DMAP) Weisburd and Green (1995)	Problem‐oriented policing strategy to control drug markets by arresting drug sellers and changing disorderly conditions. 15‐month intervention period Slow progress at treatment places caused intervention time period to be extended by 3 months.	56 drug hot spot areas identified based on ranking intersection areas with high levels of drug‐related calls and narcotics arrests, types of drugs sold, police perceptions of drug areas, and offender movement patterns.	Randomized controlled trial; control and treatment groups were each randomly allocated 28 drug hot spots within statistical blocks. Differences of differences between citizen calls during 7 month pre‐ and posttest periods, comparing control and treatment groups.
Dayton (OH) Traffic Enforcement Project Weiss and Freels (1996)	Aggressive enforcement of traffic laws to reduce more serious crimes on highway segments. 6 month intervention period. No threats to the integrity of the treatment reported.	Two roads—approximately 2 miles long; randomly selected from locations experiencing high volume of traffic located near residential and commercial properties with high levels of crime and traffic accidents over time.	Quasiexperiment; one road served as treatment, one as control. Differences in mean weekly crime were compared between treatment and control sites in addition to 3 year pretest trends within each unit using time series analysis.
San Diego (CA) Slumlords Project Clarke and Bichler‐Robertson (1998)	Slumlord forced to use competent apartment building managers to clean physical disorder that facilitated illegal drug dealing. 2‐year intervention period. No threats to the integrity of the treatment period reported.	11 multiunit apartment buildings owned by the same slumlord; slumlord identified by two officers in independent POP study.	Quasiexperiment; five properties that transitioned from a slumlord to a place manager were matched to six neighboring control properties. Pre‐ and posttest differences in year counts of arrests and calls for service at treatment properties were compared to control sites.
San Diego (CA) Place Managers Project Eck and Wartell (1998)	Property owners and building managers required to address illegal drug selling by dealing with physical and social disorder. 30‐month intervention period. No threats to the integrity of the treatment period reported	121 rental properties; represented the population of rental properties in the city over a 6 month span that experienced drug enforcement actions by the Narcotics Unit	Randomized controlled trial; rental properties were randomly assigned to either of the two treatment groups (*N* = 79) or a control group (*N *= 42) in which the police did not provide any follow up after the initial arrest. Differences of mean number of reported crimes in 6‐month intervals were compared to control group for both treatment conditions.
Spokane (WA) Public Housing Project McGarrell et al. (1999)	Community problem‐solving effort that addressed social and physical disorder problems in public housing facilities. 24‐month intervention period. No significant implementation issues noted.	Two public housing complex and the streets surrounding these locations; selected based on high levels of crime and disorder.	Quasiexperiment; one treatment complex was matched with one control complex. Difference in mean count per month over 2‐year pretest period for treatment was compared to 2‐year intervention period for control.
Midwestern City Disorder Project Novak et al. (1999)	Aggressive enforcement crackdown on public drinking, speeding and other social disorders in 10 by 12 block area 1 month. No threats to the integrity of the treatment period reported.	Two subsections of communities—10 × 12 block areas; selected by based on community complaints reported to the police, demographic characteristics, and levels of crime.	Quasiexperiment; one treatment area was matched with one control area. Difference in mean rate of crime reported per week between pre‐ and posttest levels for both areas using interrupted time series analysis.
Jersey City (NJ) Problem‐Oriented Policing Project Braga et al. (1999)	Problem‐oriented policing strategy that largely targeted social and physical disorder in violent crime hot spots. 16‐month intervention period. Initial slow progress at places caused by resistance of officers to implement intervention.	24 violent crime places identified based on ranking intersection areas with high levels of assault and robbery calls and incidents, and police and researcher perceptions of violent areas.	Randomized controlled trial; 24 places were matched into like pairs based on simple quantitative and qualitative analyses; control and treatment groups were each randomly allocated 12 places within matched pairs. Difference in differences between a number of indicators during 6‐month pre‐ and posttest periods, comparing control and treatment groups.
Oakland (CA) Beat Health Program Mazerolle et al. (2000)	Property owners and building managers required to address illegal drug selling by dealing with physical and social disorder. 5.5‐month intervention period. No threats to the integrity of the treatment period.	100 street blocks with a place on the block that was referred to the Beat Health Team as having a drug and/or blight problem.	Randomized controlled trial; control and treatment groups were each randomly allocated 50 street blocks within residential and commercial statistical blocks. Differences in differences analytic design; pre‐post time periods were 21.5 months before and 12 months after 5.5 month intervention period.
Chicago (IL) Nuisance Abatement Program Higgins and Coldren (2000)	Police engaged building owners and managers to address gangs and illegal drug selling by dealing with physical and social disorder. 4‐month intervention period. A process evaluation was conducted and no significant implementation issues were noted	121 buildings; selected based on high level of gang activity, presence of the drug trade, and geographic heterogeneity.	Quasiexperimental design; 54 buildings received treatment while 67 served as a control. Differences in counts pre‐ and postintervention between treatment and control sites compared.
Richmond (VA) Weed and Seed Initiative Smith (2001)	Community policing program that was more focused on arresting offenders than physical disorder problems in targeted zone 1‐month intervention period. No threats to the integrity of the treatment reported.	Two beats—a 5 × 10 sq. block area; selected based on level of crime and police departments assessment of neighborhood cohesiveness and political stability.	Quasiexperiment; one treatment beat area was compared to one beat. Differences in counts from 6‐month pre‐ and 6‐month posttest periods
Wales Zero Tolerance Initiative Rogers (2002)	Zero tolerance community safety strategy designed to improve quality of life by targeting vandalism and youth disorder. 12‐month intervention. Challenges with collaboration between law enforcement and social service providers influenced quality of implementation.	Three communities; no justification for the site selection was provided.	Quasiexperiment; two communities nested in the same city received treatment while the other community served as control. Differences in month and year counts of incident reports were compared between treatment and control for a 2‐year pretest period and 2‐year posttest period.
Detroit (MI) Antigang Initiative Bynum and Varano (2003)	Traditional suppression and aggressive order maintenance actions targeting gang members in 4th and 9th Precincts. 19 month intervention. The community policing component of the treatment was never fully adopted and officers did not receive as much training on gang enforcement strategies as anticipated.	Three police precincts; selected because of similar gang‐related crime problems occurring in these locations and population demographics.	Quasiexperiment; two precincts received treatment and one served as control. Time series analysis was used to compare pre, intervention, and post periods in all three precincts.
St. Louis (MO) Antigang Initiative Decker and Curry (2003)	Traditional suppression and aggressive order maintenance actions targeting gang members in 5th District. 15 month intervention. Collaboration across agencies was reported to be rare and provided an obstacle, not an asset, to the effectiveness of the treatment.	Four neighborhoods; selected because of similar gang‐related crime problems occurring in these locations and population demographics.	Quasiexperiment; two neighborhoods served as treatment while two served as control. Difference in pre‐ and posttest crime counts between treatment and control.
Jersey City (NJ) Displacement Study Weisburd et al. (2006)	Problem‐oriented policing used to target high‐rate offenders and disorderly conditions in drug and prostitution hot spots. 6‐month intervention period Burglary hot spot dropped from study due to inadequate dosage of police intervention.	Two hot spots (one drug and one prostitution) identified based on computerized mapping and database technology supplemented by police officer observations.	Quasiexperiment; observed prostitution and drug event trends were examined over a 9‐month period and adjusted for city‐wide disorder and drug call trends, respectively. Difference of means test compared pre‐ and posttest mean observed events.
Los Angeles (CA) Baldwin Safer Cities Initiative (SCI) Wagers (2007)	Broken windows policing strategy, guided by community problem‐solving concepts, to control crime in three areas. 12 month intervention. No threats to the integrity of the treatment period.	11 police districts; no justification for the site selection was provided.	Quasiexperimental; three districts received treatment and eight districts were designed controls. Changes in mean yearly crime incidents compared from pre‐post test between treatment and control
Lowell (MA) Problem‐Oriented Policing Project Braga and Bond (2008)	Problem‐oriented policing strategy that largely targeted social and physical disorder in crime hot spots. 12‐month intervention period. No threats to the integrity of the treatment reported.	34 crime and disorder hot spots identified based on spatial analyses of calls for service and supplemented by police officer and research observations.	Randomized controlled trial; 24 places were matched into like pairs based on simple quantitative and qualitative analyses; control and treatment groups were each randomly allocated 12 places within matched pairs. Differences of differences between a number of indicators during 6 month pre‐ and posttest periods, comparing control and treatment groups.
Los Angeles (CA) Safer Cities Initiative Berk and MacDonald (2010)	Place‐based policing intervention to eliminate social and physical disorder created by homeless encampments.125 week intervention. No threats to the integrity of the treatment period reported.	FIve police divisions; divisions were selected because of the high concentration of homeless and crime.	Quasiexperimental; one division received treatment while the four surrounding divisions served as controls. Regression analysis was used to estimate treatment effect on weekly time series data relative to approximately 6 years of pretest data for treatment and control divisions.
New Britain (CT) Weed and Seed Project Costanza et al. (2010)	Community policing program that was more focused on arresting offenders than physical disorder problems in targeted zone. 3 year intervention period. No threats to the integrity of the treatment period reported.	One community—337.5 sq. acres; area selected based on high level of crime	Quasiexperiment; one area served as treatment, contiguous areas as catchment, and the control areas were designated as the rest of the city. Differences in mean monthly calls for service between treatment, catchment, and control areas were compared.
Las Vegas (NV) Order Maintenance Project Pace (2010)	Specialized unit dedicated to maintaining order and enforcing misdemeanor arrests laws in targeted area. 84 day intervention. Author notes that he had no way to observe the implementation of the treatment.	Six beats; identified by high volume of calls for service, population demographics, and police intelligence collection.	Quasiexperimental; two treatment beats, two control beats located adjacent to treatment beats, and two control beats located miles away. Differences in daily means in pre and intervention time periods, pretest measures were collected on the same days in 2008 and 2009 to account for seasonal trends
Boston (MA) Smart Policing Initiative Braga et al. (2011)	Problem‐oriented policing strategy that largely targeted social and physical disorder in violent crime hot spots. 3‐year intervention period. No threats to the integrity of the treatment reported.	13 violent crime hot spots based on spatial analyses of violent street crimes and officer perceptions of place boundaries.	Quasiexperiment; 564 comparison street units were matched via propensity scores to 478 treatment street units. Growth curve regression models were used to estimate intervention effects at treatment street units relative to comparison street units over 10 year time period.
London (UK) Safe as Houses Project Agar and London Enfield Police Department (2011)	Situational prevention measures and improvements to disorderly physical conditions to reduce repeat burglaries. 241 day intervention. No threats to the integrity of the treatment reported.	One corridor of London's Enfield borough; selected because it housed a disproportionate amount of the areas burglary hot spots.	Quasiexperiment; hot spots within the corridor received treatment while the other areas of the corridor and the rest of the borough served as controls. Percent change in total number of crime reported in the pre‐ versus posttest compared to the control.
Jacksonville (FL) Hot Spots Policing Project Taylor et al. (2011)	Problem‐oriented policing strategy that largely targeted social and physical disorder in violent crime hot spots. 90‐day intervention period. No threats to the integrity of the treatment reported.	83 violent crime hot spots identified based on spatial analyses of incidents and calls for service.	Randomized controlled trial; 83 places were randomly allocated in statistical blocks to problem‐oriented treatment (22), direct‐saturation patrol treatment (21), and control (40) conditions. Differences in differences between a number of violent and property crime indicators during 1‐year pretest and 90 day posttest periods, comparing control and experimental groups.
Redlands (CA), Ontario (CA), and Colton (CA) Suburbs Broken Windows Project Weisburd et al. (2012)	Broken windows policing strategy that targeted social and physical disorder at high‐crime street segments in three cities. 6‐month intervention period. A detailed process evaluation was conducted that found minor implementation issues such as treatment decay toward the end of the intervention but overall the authors noted no major limitations to the overall effectiveness of the program.	108 street segments across three cities; selected based on disorder and Part I offense calls for service in area. Number of street segments selected from each city was predetermined based on city's population.	Randomized experiment; 54 street segments received treatment and 54 street segments served as control, block‐randomization used. Differences in mean monthly calls for service pre‐post test.
Lowell (MA) Smart Policing Initiative (SPI) Bond and Hajjar (2013)	Community problem‐solving intervention to address social and physical disorder in property crime hot spots. 16‐month intervention. No threats to the integrity of the treatment reported.	24 hot spots; selected based on combination of hot spots analysis and police intelligence gathering.	Quasiexperiment; 12 treatment hot spots were matched to 12 control hot spots. Changes in sector counts for the intervention were compared to pretest counts across treatment and control sites.

**Table 4:** Results of disorder policing randomized experiments and quasiexperiments

Study	Crime outcomes	Other outcomes	Displacement/diffusion
Newark (NJ) Signs of Crime Program Pate and Skogan (1985a)	Statistically significant decreases in total Part I crimes (−22.2%), burglaries (−38.8%), and personal crimes (−29.3%).	Multiple surveys were conducted on aspects of the police–community relation and individual fear of crime. Residents of the treatment area reported high levels of victimization, lower levels of satisfaction, and lower levels of police aggressiveness.	Not measured
Newark (NJ) Community Policing Program Pate and Skogan (1985b)	Statistically significant reduction in total crimes (−23.7%), auto theft (38.1%), and outdoor crimes (−34.5%) was found.	Multiple surveys were conducted on a various aspects of the police–community relation and individual fear of crime. Residents reported lower levels of perceived disorder and higher levels of satisfaction with the police.	Not measured
Unspecified Southeastern City Foot Patrol Project Esbensen (1987)	Decreases in quality of life offenses but no statistically significant reduction in crime trend.	A survey was conducted to gauge levels of police professionalism, levels of support to the police, and police/community relations. The author did not find changes in perceptions of the police based on the intervention.	Catchment areas for both the treatment and control areas were constructed ‐no specification on the size of the areas was provided. Difference in counts during the intervention period suggest a displacement effect.
New York (NY) Community Patrol Officer Program (CPOP) McElroy et al. (1990)	No consistent support for a treatment effect was found.	Surveys were filled out by community members, CPO officers, and police command staff to gauge each stakeholders experience with the initiative. Overall all stakeholders viewed the initiative favorably.	Not measured
Jersey City (NJ) DMAP Weisburd and Green (1995)	Statistically significant reductions in disorder calls for service in treatment drug markets relative to control drug markets. No change in violent and property crime calls.	None	Examined displacement and diffusion effects in two‐block catchment areas surrounding the treatment and control drug places and replicated the drug market identification process. Little evidence of displacement; analyses suggest modest diffusion of benefits for disorder.
Dayton (OH) Traffic Enforcement Project Weiss and Freels (1996)	The only significant treatment effect was a decrease in special arrests but the direction was the opposite of what was hypothesized.	Traffic accidents in experimental location. No significant treatment effect observed.	Not measured
San Diego (CA) Slumlords Project Clarke and Bichler‐Robertson (1998)	No tests of statistical significance were conducted. Authors conclude a treatment effect was observed based on observations of decreases in calls for service and arrests compared to control.	None	Not measured
San Diego (CA) Place Managers Project Eck and Wartell (1998)	60% reduction in crime for meeting‐treatment locations, the letter‐treatment group did not experience significant reductions.	None	Not measured
Spokane (WA) Public Housing Project McGarrell et al. (1999)	No significant reduction of crime between treatment and control sites.	Several other outcomes were measured; a reduction in residents fear of crime was observed, no change in perceptions of neighborhood, signs of guardianship increased, no reduction in signs of disorder for most buildings.	Not measured
Unknown Midwestern City Disorder Project Novak et al. (1999)	No significant treatment effect observed.	None	Examined effects in catchment areas three to four streets large for both the treatment and control sites No displacement or diffusion effect observed.
Jersey City (NJ) POP at Violent Places Braga et al. (1999)	Statistically significant reductions in total calls for service and total crime incidents. All crime categories experienced varying reductions; statistically significant reductions in street fight calls, property calls, narcotics calls, robbery incidents, and property crime incidents.	Observation data revealed that social disorder was alleviated at 10 of 11 treatment places relative to control places. Nonexperimental observation data revealed that physical disorder was alleviated at 10 of 11 treatment places. Nonexperimental interviews with key community members in target locations suggest no noteworthy improvements in citizen perceptions of places.	Examined displacement and diffusion effects in two‐block catchment areas surrounding the treatment and control violent places. Little evidence of immediate spatial displacement or diffusion
Oakland (CA) Beat Health Program Mazerolle, Price, and Roehl (2000)	Statistically significant reductions in drug calls in treatment blocks relative to control blocks; no statistically significant differences in other call types.	None	Examined displacement and diffusion effects in 500 foot radii catchment areas surrounding the treatment and control street blocks. Analyses of catchment areas suggested an overall diffusion of crime control benefits for treatment catchment areas relative to control catchment areas.
Chicago (IL) Nuisance Abatement Program Higgins and Coldren (2000)	Statistically significant reductions in narcotics crime incidents.	Other crime outcomes explored, a general treatment effect was found across these measures.	Not measured
Richmond (VA) Weed and Seed Initiative Smith (2001)	A statistically significant 92% reduction in reported crime occurred in treatment area.	None	7 months of data was analyzed for 3 surrounding zones. No displacement effects observed.
Wales Zero Tolerance Initiative Rogers (2002)	Treatment was associated with a 6.3% increase in total crime incidents.	Pre and post treatment surveys were given to community members to measure fear of crime. Overall, the initiative did not consistently demonstrate reductions in fear of crime.	No discussion of how catchment areas were measured. No evidence of crime displacement.
Detroit (MI) Anti‐Gang Initiative Bynum and Varano (2003)	A statistically significant reduction in crime incidents observed in only one treatment precinct compared to control area.	None	Not measured
St. Louis (MO) Anti‐Gang Initiative Decker and Curry (2003	No significant treatment effect observed.	None	Not measured
Jersey City (NJ) Displacement and Diffusion Study Weisburd, Wickoff, Ready, Eck, Hinkle, and Gajewski (2006)	Statistically significant 45% reduction at the targeted prostitution location. Statistically significant 58% reduction at the targeted drug crime location.	Ethnography and interviews with arrested offenders confirmed that offenders did not displace from targeted locations into surrounding areas.	Examined displacement and diffusion effects in one and two block catchment areas surrounding targeted locations. Analyses revealed significant diffusion of crime control benefits.
Los Angeles (CA) Baldwin Safer Cities Initiative (SCI) Wagers (2007)	Significant treatment effect observed	Citizen complaints and use of force grievances in areas; no significant differences found after the implementation of the treatment.	Not measured
Lowell (MA) Policing Crime and Disorder Hot Spots Project Braga and Bond (2008)	Statistically significant reductions in total calls for service. All crime categories experienced varying reductions; statistically significant reductions in street fight calls, property calls, narcotics calls, robbery incidents, and property crime incidents.	Observation data revealed that social disorder was alleviated at 14 of 17 treatment places relative to control places. Observation data revealed that physical disorder was alleviated at 13 of 17 treatment places relative to control places. Pre‐ and posttest interviews with key community members in treatment and control locations suggest that disorder problems were positively impacted.	Examined displacement and diffusion effects in two‐block catchment areas surrounding the treatment and control violent places. No evidence of immediate spatial displacement or diffusion.
Los Angeles (CA) Safer Cities Initiative Berk and MacDonald (2010)	Significant reductions in property crime, violent crime, and nuisance crime incidents.	None	Examined displacement and diffusion by respecifying the models to have the control areas serve as catchment areas. No displacement effects were detected while diffusion effects were found intermittently.
New Britain (CT) Weed and Seed Project Costanza et al. (2010)	No significant differences in calls for service.	Exploratory spatial data analysis (ESDA); Locations of arrests and calls for assistance were mapped and analyzed. ESDA and weighted displacement quotient analyses do not indicate proximity effects, local indicators of spatial association (LISA) maps show noteworthy changes in the spatial clustering of arrest activity over time.	Examined displacement and diffusion effects in catchment area with identical size as treatment area (337.5 sq. acres) using differences in mean monthly calls for service, arrests and mapping techniques. Displacement was not found for calls for service.
Las Vegas (NV) Order Maintenance Policing Project Pace (2010)	Only significant treatment effect was on one incident measure.	Self initiated field activity data were collected in treatment and control areas with a significant increase noted.	Not measured
Boston (MA) Safe Street Teams Program Braga et al. (2011)	Statistically significant 14% reduction in violent crime incidents.	None	Examined displacement and diffusion effects in two‐block catchment areas surrounding the treatment and control street units. No evidence of immediate spatial displacement or diffusion.
London (UK) Operation “Safe as Houses” Program Agar/London Enfield Police Department (2011)	A 46.7% decrease in burglaries associated with the treatment while the controls only experienced 1.8% and 7.2%.	None	Examined displacement and diffusion effects in areas of the corridor not targeted for treatment as catchment areas. Simple pre‐post percent change calculates indicate a diffusion of benefits for catchment area but some evidence for displacement in other portions of the borough.
Jacksonville (FL) Policing Violent Crime Hot Spots Program Taylor et al. (2011)	Problem‐oriented policing generated statistically significant 33% reduction in street violence. Direct‐saturation patrol did not generate any statistically significant reductions.	None	Examined displacement and diffusion effects in 500 feet buffer zones surrounding the treatment and control violent places. Evidence of immediate spatial displacement associated with problem‐oriented policing intervention.
Redlands (CA), Ontario (CA), and Colton (CA) Suburbs Broken Windows Project Weisburd et al. (2012)	No significant reductions in disorder or any crime measures.	Surveys of residents in treatment and control areas on fear of crime, police legitimacy, collective efficacy, and perceptions of crime. A significant increase in perceptions of physical disorder but no other significant changes in outcomes were measured as the result of the treatment.	Not measured
Lowell (MA) Lowell Smart Policing Initiative (SPI) Bond and Hajjar (2013)	A 16.9% reduction in treatment sites occurred compared to a 5.3% reduction in control sites.	None	Not measured

A majority of the eligible studies concluded that disorder policing programs generated statistically significant crime control benefits in the treatment areas relative to the control areas. Overall, 19 of the 30 tests (63.6%) of disorder policing interventions reported noteworthy crime control gains associated with the intervention. The largest crime control effects reported by randomized controlled trials were observed by the San Diego Place Managers Project (60% reduction in total incidents reported; Eck and Wartell, 1998), with the Jersey City Displacement and Diffusion Study observing the next two highest (58% reduction in drug crime incidents and 45% reduction in prostitution; Weisburd et al., 2006).

The largest crime control effects reported by quasiexperiments were observed in the Richmond Weed and Seed Initiative (92% reduction in total incidents reported; Smith, 2001) and Operation Safe as Houses (46.7% reduction in burglary incidents; London‐Enfield Police Department, 2011). The Los Angeles Suburbs Broken Windows Project (Weisburd et al., 2012) was the only one of eight randomized controlled trials that did not report crime reductions. Seven of the 22 quasiexperiments did not report crime reductions; only two of these were community problem solving interventions (New York CPOP and Spokane Public Housing Project). The five studies that focused on aggressive enforcement interventions were the Dayton Traffic Enforcement Project, Midwestern City Disorder Project, Wales Zero Tolerance Initiative, St. Louis Antigang Initiative, and the New Britain Weed and Seed Project.

Fifteen of the 30 tests examined whether disorder policing efforts were associated with crime displacement or diffusion of crime control benefits (see Table 4). Prior to a discussion of the research findings, it must be noted that it is quite difficult to detect displacement effects, because the potential manifestations of displacement are varied. As Barr and Pease (1990) suggest, “if, in truth, displacement is complete, some displaced crime will fall outside the areas and types of crime being studied or be so dispersed as to be masked by background variation… no research study, however massive, is likely to resolve the issue” (p. 293). Diffusion effects are likely to be as difficult to assess. All 15 tests were limited to examining immediate spatial displacement and diffusion effects; that is, whether disorder policing efforts in targeted areas resulted in crime “moving around the corner” or whether these proximate areas experienced unintended crime control benefits.

Our review suggests that diffusion of crime control effects and no displacement effects were more likely to be observed than crime displacement effects. Only four of the 15 studies reported immediate spatial displacement of crime into areas surrounding the targeted locations. The tests that reported crime displacement effects were in the Southeastern City Foot Patrol Project (Esbensen, 1987), New Britain Weed and Seed Project (Costanza et al., 2010), and Jacksonville Problem‐Oriented Policing at Violent Crime Hot Spots experiment (Taylor et al., 2011). However, five tests suggested possible diffusion effects associated with the disorder policing interventions. Some of the tests that reported statistically significant diffusion effects were in the Jersey City DMAP experiment (Weisburd & Green, 1995), Oakland Beat Health study (Mazerolle et al., 2000), and the Jersey City Displacement and Diffusion Study (two tests: buffer zones surrounding the targeted prostitution hot spot and the targeted drug hot spot; Weisburd et al., 2006). The remaining six studies did not report significant displacement or diffusion effects.

#### 7.1.4 Study Implementation

Most of the eligible studies did not report any noteworthy implementation problems. Seven studies (25.0% of 28), however, did report potential threats to the integrity of the treatment. The New York CPOP (McElroy et al. 1990) and Detroit Antigang Initiative (Bynum & Varano, 2003) both reported difficulty with officers adopting the community policing techniques outlined in the treatment. The Wales Zero Tolerance Initiative (Rogers, 2002) and St. Louis Antigang Initiative (Decker & Curry, 2003) also experienced challenges in facilitating the interagency collaboration that was a key feature of the intervention.

The Jersey City DMAP experiment (Weisburd & Green, 1995, p. 721) and Jersey City POP at Violent Places experiment (Braga, 1997, pp. 107–142) reported instances where the treatments were threatened by program implementation subversion by the participants. The officers charged with preventing crime at the treatment hot spots were resistant to participating in the programs and this resulted in low levels of treatment during the early months of both experiments. In the Jersey City DMAP experiment, this situation was remedied by providing a detailed crackdown schedule to the Narcotics Squad commander and extending the experiment from 12 to 15 months. This problem was remedied in the Jersey City POP experiment by changing the leadership of the POP unit, developing an implementation accountability system, providing additional training in the problem‐oriented policing approach, and through other smaller adjustments. In the Jersey City Displacement and Diffusion Study, focused police attention was originally applied to three crime hot spots; unfortunately, the Police Foundation research team detected that the intervention was not being applied with an adequate dosage in the burglary hot spot and, as such, dropped the location from the evaluation (Weisburd et al. 2006). Of course, these implementation problems are not unique to disorder policing experiments and quasiexperiments; many well‐known criminal justice field experiments have experienced and successfully dealt with methodological difficulties.^2^

#### 7.1.5 Risk of bias in included studies

Table 5 presents our assessment of risk of bias in the 28 included disorder policing studies. We assessed the level of risk of bias along six sources of potential bias for each study (“Low” or “High”), or if a study was not clear on whether the bias was present or not (“Unclear”). The dimensions of bias assessed were: (a) To what extent was randomization absent in the allocation of study units? (b) How much did the assignment sequence stray from proper randomization protocol? (c) How dissimilar were treatment and control units at the baseline? (d) What level of contamination was present in the study? (e) To what degree did this study engage in selective reporting? (f) How much did other reported risks of bias threaten the integrity of this study?

**Table 5:** Assessment of risk of bias in eligible disorder policing studies

Study (author(s), year)	Random allocation^a^	Randomization process^b^	Selection^c^	Protection from contamination^d^	Nonreporting^e^	Other bias^f^
Pate and Skogan (1985a)	High	High	Low	Low	Low	Low
Pate and Skogan (1985b)	High	High	Low	Low	Low	Low
Esbensen (1987)	High	High	Low	Low	Low	Low
McElroy et al. (1990)	High	High	Low	Low	Low	High
Weisburd and Green (1995)	Low	Low	Low	Low	Low	High
Weiss and Freels (1996)	High	High	Unclear	Low	Low	Low
Clarke and Bichler‐Robertson (1998)	High	High	Low	Low	Low	Low
Eck and Wartell (1998)	Low	Low	Low	Low	Low	Low
Novak et al. (1999)	High	High	Low	Low	Low	Low
Braga et al. (1999)	Low	Low	Unclear	Low	Low	High
McGarrell et al. (1999)	High	High	Low	Low	Low	Low
Mazerolle et al. (2000)	Low	Low	Low	Low	Low	Low
Higgins and Coldren (2000)	High	High	High	Low	Low	Low
Smith (2001)	High	High	Low	Low	Low	Low
Rogers (2002)	High	High	High	Low	Low	High
Bynum and Varano (2003)	High	High	Low	Low	Low	High
Decker and Curry (2003)	High	High	Low	Low	Low	High
Weisburd et al. (2006)	High	High	High	Low	Low	High
Wagers (2007)	High	High	High	Low	Low	Low
Braga and Bond (2008)	Low	Low	Unclear	Low	Low	Low
Costanza et al. (2010)	High	High	High	Low	Low	Low
Pace (2010)	High	High	Low	Low	Low	Unclear
Berk and MacDonald (2010)	High	High	Low	Low	Low	Low
Braga et al. (2011)	High	High	Low	Low	Low	Low
Taylor et al. (2011)	Low	Low	Low	Low	Low	Low
Enfield Police Department (2011)	High	High	High	Low	Low	Low
Weisburd et al. (2012)	Low	Low	Low	Low	Low	Low
Bond and Hajjar (2013)	High	High	High	Low	Low	Low
“High” totals	21	21	7	0	0	7
% of *N* = 28 studies	75.0%	75.0%	25.0%	0.0%	0.0%	25.0%

^a^ To what extent was randomization absent in the allocation of study units?

^b^ How much did the assignment sequence stray from proper randomization protocol?

^c^ How dissimilar were treatment and control units at the baseline?

^d^ What level of contamination was present in the study?

^e^ To what degree did this study engage in selective reporting?

^f^ How much did other reported risks of bias threaten the integrity of this study?

All seven randomized controlled trials included in this review used credible methods for randomization and did not report any issue in the implementation of the randomization scheme implemented. However, there were some limitations to the internal validity of the included studies. Only two‐thirds of eligible studies (*N *= 18, 64.3%) provided direct evidence (usually in the form of a table that presented balanced outcomes and descriptive variables) that the treatment and control units were similar at the baseline measurement period. Another three studies (10.7%) provided descriptions of methods, such as block randomization (e.g., Braga et al. 1999) and simple matching exercises (e.g., Weiss & Freels, 1996), that create balanced treatment and control groups but did not provide clear evidence that the described techniques actually achieved balance. Seven studies (25.0%) used treatment and control units that were not the same. For instance, the Jersey City Displacement and Diffusion Study compared crime outcomes in the targeted areas relative to crime outcomes in the rest of the city.

All included studies did not report any evidence of contamination of control conditions during the intervention period. None of the included studies reported evidence suggestive that the evaluators were only selecting those crime types that showed an effect. Finally, seven studies presented other evidence of possible bias; as described above, these studies noted relatively mild program implementation problems. As such, we can conclude that the internal validity of the included studies was generally high. There were variations in the overall strength of the research designs used by included studies: seven studies (representing seven independent tests) used randomized controlled trials and 21 studies (involving 23 independent tests) used quasiexperimental designs. As such, we conducted sensitivity analyses that tested the moderating effects of research design on the relationship between disorder policing programs and crime outcomes.

### 7.2 Synthesis of results

#### 7.2.1 Meta‐analysis of effects of disorder policing on crime

**Figure 1:**
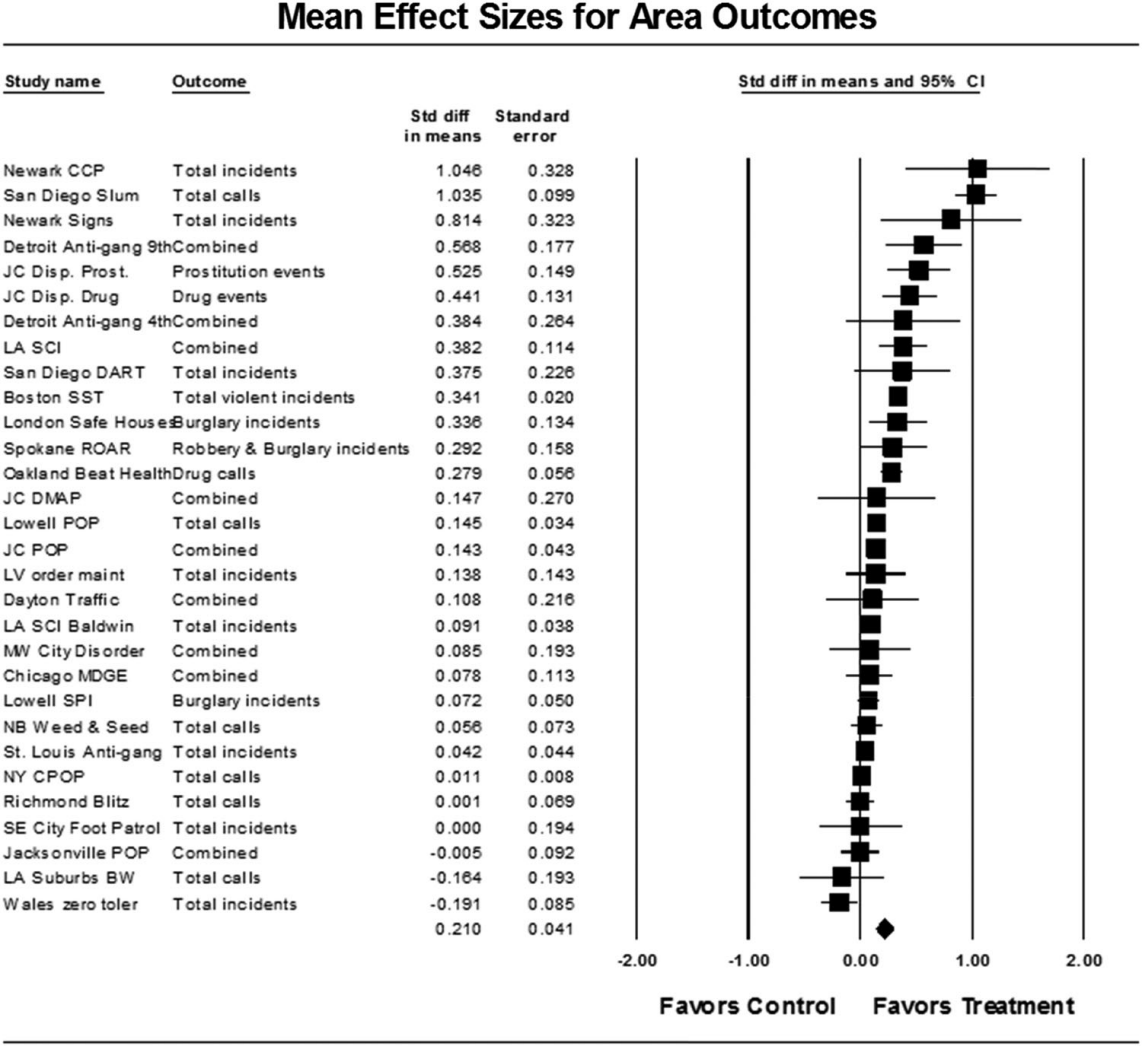
Random effects meta‐analysis of the overall effects of policing disorder programs on crime

Our meta‐analysis used a random effects model to estimate the overall mean effect of disorder policing on crime outcomes.^3^ Using the overall mean effect size from each study for 30 main effects tests, the forest plots in Figure 1 shows the standardized difference in means between the treatment and control or comparison conditions (effect size) plus its 95% confidence interval. Points plotted to the right of zero indicate a treatment effect; in this case, the test showed a reduction in crime or disorder. Points to the left of zero indicate a backfire effect where control conditions improved relative to treatment conditions. The meta‐analysis suggests a statistically significant effect in favor of policing disorder strategies. The overall effect size is *d* = 0.210 (*p *< .05; 95% CI [0.130, 0.290]), suggesting a modest but meaningful impact on crime (see Cohen, 1988).

**Figure 2:**
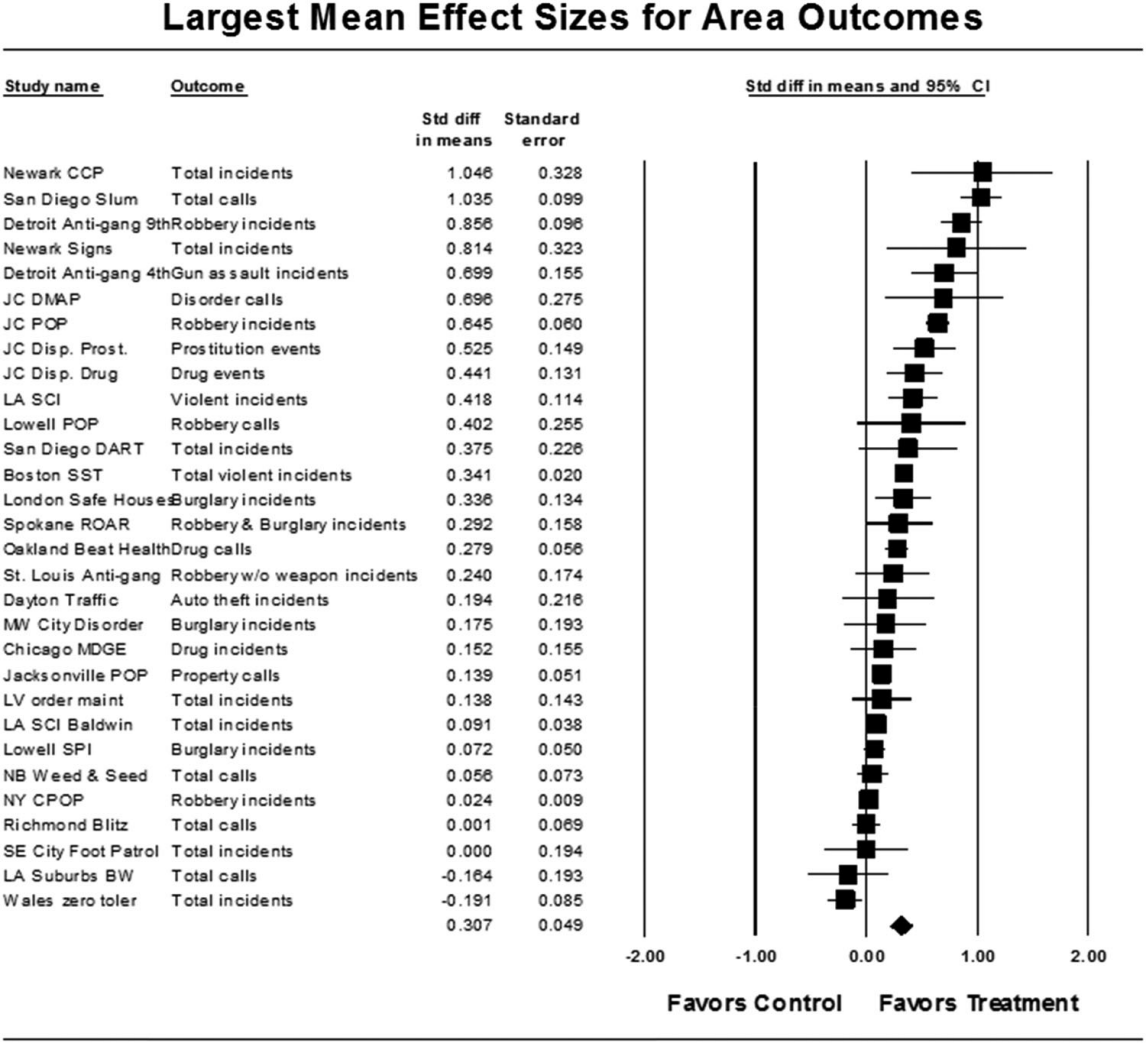
Random effects meta‐analysis of the largest effects of policing disorder programs on crime

**Figure 3:**
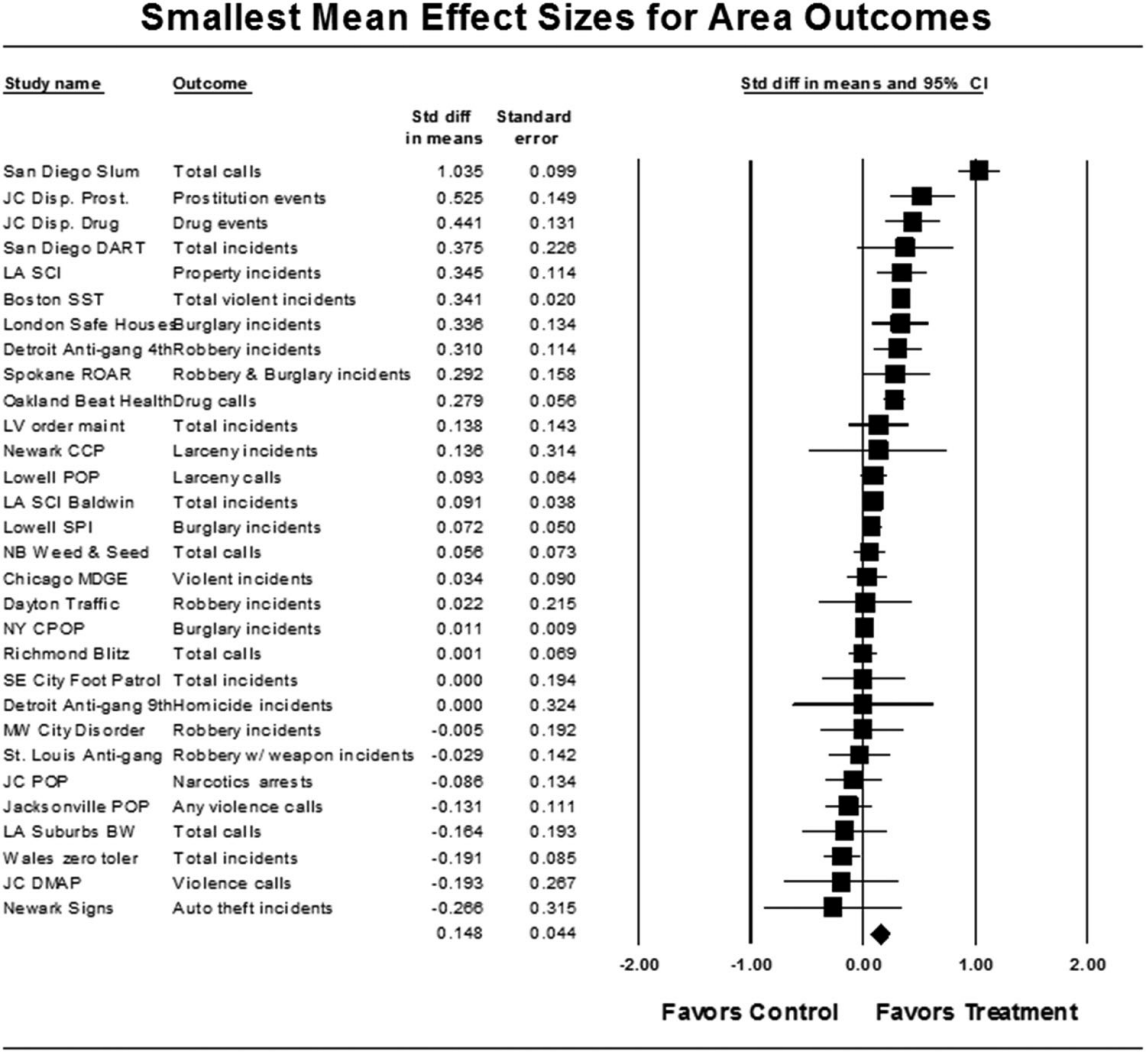
Random effects meta‐analysis of the smallest effects of policing disorder programs on crime

Twenty‐six tests reported effect sizes that favor treatment conditions over control conditions. The Newark Community Policing Program quasiexperiment, San Diego Slumlords Project quasiexperiment, and Newark Signs of Crime Program tests reported the largest statistically significant effect sizes (*p *< .05), while the Los Angeles Baldwin Safer Cities Initiative quasiexperiment reported the smallest statistically significant effect size (*p* < .05). The forest plots in Figures 2 and 3 present the meta‐analyses of the largest and smallest effect sizes for each study, respectively.^4^ For the largest effect size meta‐analysis, the overall effect size is moderate (0.307) and statistically significant (*p *< .05; 95% CI [0.210, 0.404]). For the smallest effect size meta‐analysis, the overall effect size is small (0.148) and statistically significant (*p *< .05; 95% CI [0.062, 0.233]). Table 6 presents mean effect sizes for the effects of disorder policing programs on violent crime, property crime, and drug/disorder offense outcomes. Disorder policing programs produced statistically significant (*p* < .05) positive mean effect sizes for drug/disorder offense outcomes (0.266; 95% CI [0.168, 0.365]), violent crime outcomes (0.231; 95% CI [0.098, 0.365]), and property offense outcomes (0.192; 95% CI [0.092, 0.293]).

**Table 6:** The effects of disorder policing programs on specific types of crime outcomes

Crime type	*K*	Mean effect size	95% CI
Violent	15	0.227*	0.098, 0.365
Property	16	0.187*	0.092, 0.293
Disorder/drug	9	0.266*	0.168, 0.365
Total	30	0.210*	0.130, 0.290

*Note*: Random effects meta‐analysis models used in all reported effect sizes.

* *p* < .05.

**Figure 4:**
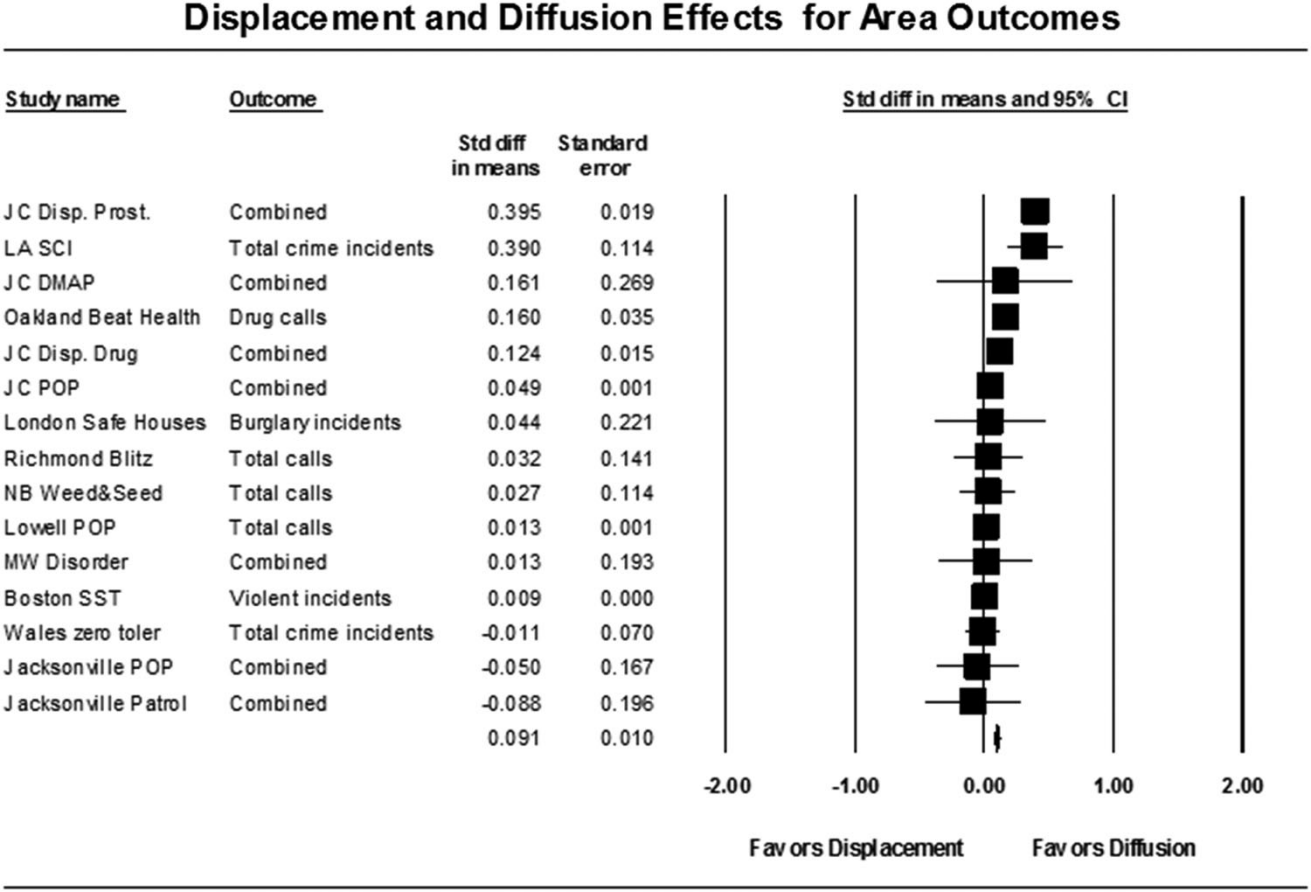
Random effects meta‐analysis of crime displacement and diffusion impacts

A random effects model examined crime displacement and diffusion impacts for 15 policing disorder tests that measured these outcomes. The forest plot in Figure 4 shows that the overall effect size favors a diffusion of crime control benefits impact over a crime displacement effect impact; the overall effect size is small but statistically significant (0.091, *p *< .05; 95% CI [0.072, 0.111).^5^ The largest effect sizes suggesting diffusion of crime control benefits were generally estimated from hot spots policing program evaluations where policing disorder interventions were applied to very small high‐activity crime places. A prior Campbell review found that hot spots policing programs were more likely to generate diffusion impacts rather than crime displacement impacts (Braga et al. 2012).

**Figure 5:**
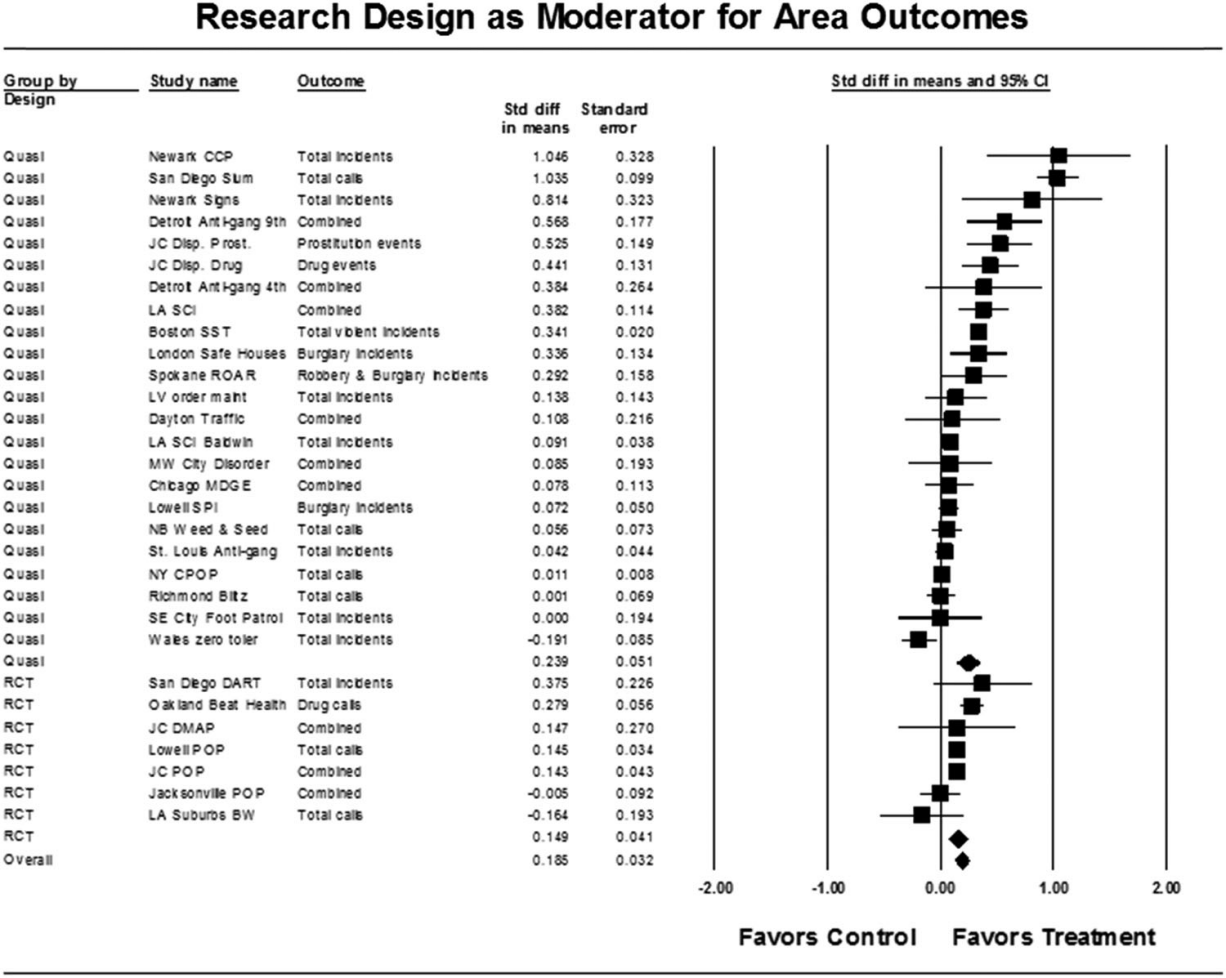
Random effects meta‐analysis of the effects of different types of program evaluation designs on crime

Given the important distinction in methodological quality between the randomized controlled trials and quasiexperimental evaluation studies, we also examined research design as a moderator variable. Figure 5 presents a random effects model examining the two different types of evaluation designs included in this review.^6^ Consistent with prior research suggesting that weaker designs tend to report stronger effects in crime and justice studies (Weisburd, Lum, & Petrosino, 2001; Welsh, Peel, Farrington, Elffers, & Braga, 2011), the quasiexperimental designs were associated with a somewhat larger within‐group mean effect size (*d* = 0.239, *p* < .05; 95% CI [0.139, 0.339]) relative to the experimental designs (*d* = 0.149, *p* < .05; 95% CI [0.069, 0.230]). When research design type was included as a moderator, the meta‐analysis estimated a more modest overall effect size (*d* = 0.185, *p* < .05; 95% CI [0.122, 0.247]). This does not mean that quasiexperimental studies cannot be of high quality, but only that there is evidence that quasiexperimental designs in disorder policing evaluations seem likely to overstate outcomes as contrasted with randomized experiments. However, the purported relationship between quasiexperimental designs and larger effect sizes has not been universally found (see, e.g., Shadish and Ragsdale, 1996).

#### 7.2.2 Program type as effect size moderator

Our review documented that disorder policing programs have adopted community problem‐solving programs and aggressive order maintenance programs to control crime. Community problem‐solving programs would use a combination of collaborations with community stakeholders and problem analyses to identify creative solutions to removing disorder from places. Aggressive order maintenance programs often relied exclusively on the intensive enforcement of social disorder as the singular treatment to reduce disorder in communities. There can be, of course, some overlap between the enforcement interventions employed by the community problem‐solving programs and the actions taken by the aggressive order maintenance programs. However, these two general types of programs represent fundamentally different orientations in dealing with physical and social disorder.

**Figure 6:**
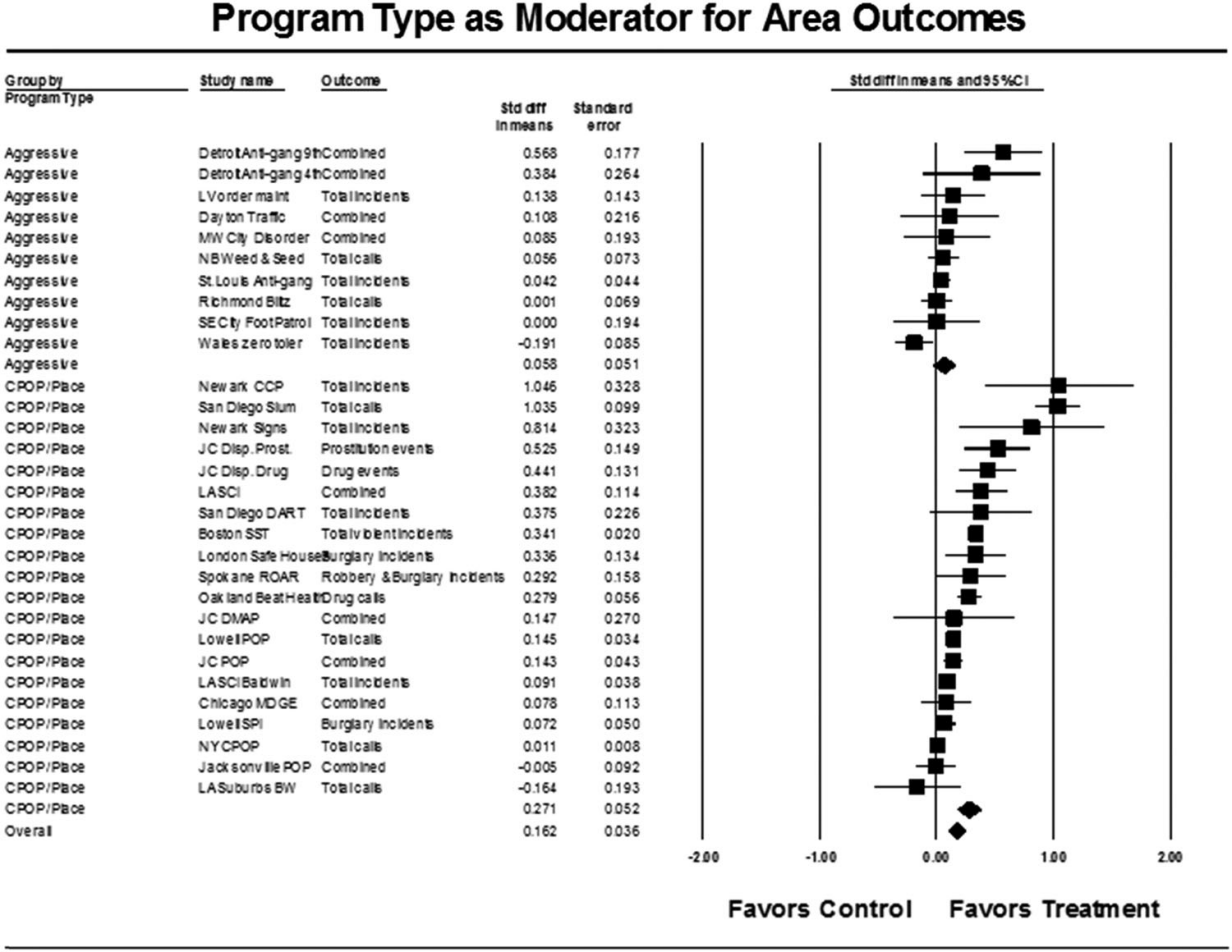
Random effects meta‐analysis of the effects of different types of policing disorder programs on crime

Moderator variables help to explain and understand differences across studies in the outcomes observed. Program type could be an influential moderator of the observed effect sizes in our meta‐analysis. Figure 6 presents a random effects model examining the two different program types: community problem‐oriented policing and aggressive order maintenance policing.^7^ Our meta‐analysis shows that community problem‐oriented policing programs produce a larger overall mean effect size (0.271, *p* < .05; 95% CI [0.169, 0.373]), which is almost five times the size of the aggressive order maintenance policing overall mean effect size (0.058, *ns*; 95% CI [−0.041, 0.158).

#### 7.2.3 Publication bias

Publication bias presents a serious challenge to any review of evaluation studies (Rothstein, 2008). Our extensive search procedures, the use of an information retrieval specialist (Phyllis Schultze), and the mobilization of an extensive network of police scholars made it unlikely that relevant unpublished works would remain hidden from this review. Indeed, nearly one‐third (9 of 30, 30.0%) of the included tests of disorder policing programs came from “grey literature” sources such as published reports, unpublished reports, and dissertations. We used the trim‐and‐fill procedure (Duval & Tweedie, 2000) to estimate the effect of potential data censoring, such as publication bias, on the outcome of the meta‐analyses. The diagnostic funnel plot is based on the idea that, in the absence of bias, the plot of study effect sizes should be symmetric about the grand mean effect size. If there is asymmetry, the trim‐and‐fill procedure imputes the missing studies, adds them to the analysis, and then recomputes the grand mean effect size.

**Figure 7:**
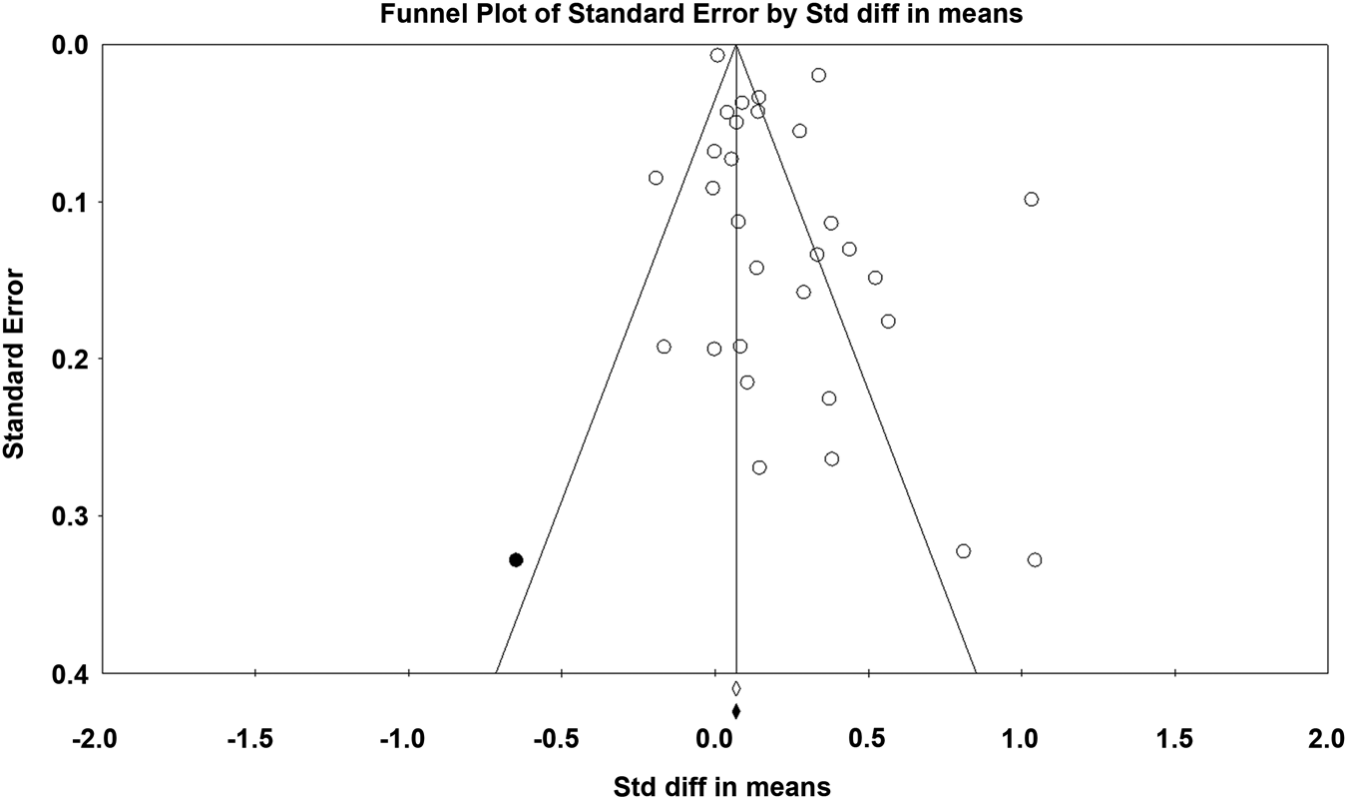
Funnel plot for all eligible studies with imputed studies from trim‐and‐fill analysis. Empty circles are the original studies. The filled‐in circle indicates the imputed study from the trim‐and‐fill analysis

A visual inspection of the resulting funnel plot in Figure 7 indicates very minor asymmetry, with one study added to create symmetry. This altered marginally the overall mean effect size, from *d* = 0.210 (95% CI [0.129, 0.289]) to *d* = 0.199 (95% CI [0.120, 0.279]). Indeed, the 95% confidence intervals overlap substantially, suggesting that the mean effect sizes approximate one another and, importantly, publication bias does not appear to alter our findings.

## 8 DISCUSSION

### 8.1 Summary of main results

The results of our systematic review and meta‐analysis suggest that disorder policing generates noteworthy crime control gains, yielding consistent crime reduction effects across a variety of violent, property, drug, and disorder outcome measures. When measured, the disorder policing interventions did not result in immediate spatial displacement of crime into proximate areas. Rather, our analyses suggest that surrounding areas experience diffusion of crime control benefits from disorder policing interventions implemented in targeted areas. Importantly, of the two main strategies of disorder policing, community and problem‐solving—not aggressive, order maintenance—is associated with reductions in crime.

These findings provide support for police paying attention to social and physical disorder when seeking to reduce more serious crimes in neighborhoods. Indeed, beyond disorder policing, these general ideas support key strategies and tactics employed by a wide range of recent police innovations, such as community policing, problem‐oriented policing, third‐party policing, and hot spots policing (see Weisburd & Braga, 2006). Police departments should continue to engage policing disorder strategies as part of their portfolio of strategies to reduce crime.

### 8.2 Overall completeness and applicability of evidence

Given the ubiquity of disorder policing in many nations, the findings produced in this review have widespread relevance to the field of policing and crime prevention. Most of the included disorder policing studies were completed in the United States (28 studies); however, two evaluations were implemented in the United Kingdom suggesting possible general applicability of disorder policing across varying contexts. Nevertheless, police departments in countries beyond the United States should be cautious in adopting these programs as further evaluation research is needed to confirm the crime control value of this approach in other settings.

### 8.3 Quality of the evidence

The overall quality of evidence presented in this review is robust. Randomized controlled trial designs were used in almost one‐third of eligible studies and among the quasiexperimental studies, many used rigorous evaluation methods. Positive crime control findings were observed for both experimental and quasiexperimental research designs. Nearly two‐thirds of eligible studies demonstrated that treatment and control units were similar at the baseline measurement period. There was no evidence that authors of eligible studies engaged in selective reporting of crime outcomes. Furthermore, evidence of contamination of treatment was absent in all of the eligible studies.

### 8.4 Limitations and potential biases in the review process

Outcome measures by studies included in this review relied exclusively on official records and did not include measures of self‐report victimization. As such, our findings could be biased by police decision‐making processes and other biases.

### 8.5 Agreements and disagreements with other studies or reviews

The results of this systematic review support the general assertion that police can be effective in preventing crime (Skogan & Frydl, 2004) and the results are consistent with other Campbell Collaboration reviews on hot spots policing (Braga, 2007; Braga et al. 2012), focused deterrence policing (Braga & Weisburd, 2012), problem‐oriented policing (Weisburd et al., 2008), and crime displacement (Bowers et al., 2011). Our conclusion that disorder policing is effective in controlling crime is consistent with some narrative reviews (e.g., Kelling & Coles, 1996), but diverges from other narrative reviews (Harcourt, 2001).

## 9 AUTHORS' CONCLUSIONS

### 9.1 Implications for practice and policy

More than 30 years of evaluation research on the impact of disorder policing strategies on crime has produced a large body of studies characterized by an array of positive, null, and negative effects. Unfortunately, scholars and policy analysts have not attempted to synthesize the findings of these empirical studies in a systematic way. Prior narrative reviews of this body of research privileged the findings of particular studies over others and, as a result, produced divergent conclusions on the crime control impact of disorder policing. For instance, in a published debate, Columbia University law professor Bernard Harcourt concluded that there was “no good evidence” that disorder policing reduces serious crime, while University of Michigan public policy professor David Thacher suggested that there were some indications that disorder policing may positively impact crime rates (Harcourt & Thacher, 2005, p. 15). In contrast to narrative reviews, systematic reviews and meta‐analyses provide rigorous methodologies and statistical procedures to summarize, integrate, and interpret the overall findings of a well‐defined set of scholarly works.

The findings of this review suggest that disorder policing strategies do generate crime control benefits. Perhaps of greatest interest to police leaders and policymakers alike is that the types of strategies used by police departments to control disorder seem to matter. Aggressive order maintenance strategies that target individual disorderly behaviors do not generate significant crime reductions. In contrast, community problem‐solving approaches that seek to change social and physical disorder conditions at particular places produce significant crime reductions. These findings suggest that, when considering a policing disorder approach, police departments should adopt a “community co‐production model” rather than drift toward a zero‐tolerance policing model, which focuses on a subset of social incivilities, such as drunken people, rowdy teens, and street vagrants, and seeks to remove them from the street via arrest (Taylor, 2001). In devising and implementing appropriate strategies to deal with a full range of disorder problems, police must rely on citizens, city agencies, and others in numerous ways. As Taylor (2001) suggests, incivility reduction is rooted in a tradition of stable relationships with the community and responsiveness to local concerns. A sole commitment to increasing misdemeanor arrests stands a good chance to undermine relationships in low income, urban communities of color, where coproduction is most needed and distrust between the police and citizens is most profound (Brunson, 2007; Skogan & Frydl, 2004).

### 9.2 Implications for research

The effect size difference noted by our analysis of the program type moderator variable should be regarded as a new hypothesis to be subjected to further testing rather than an established conclusion. Disorder problems, and the police programs designed to ameliorate disorderly conditions, are highly contextualized to local conditions. Moderator variables cannot be assumed to capture statistically independent conditions and, as such, great care must be taken when interpreting the relationship between moderator variables and effect sizes in meta‐analysis (Lipsey, 2003). Our broad categorization of disorder policing programs into “community problem solving” and “aggressive order maintenance” interventions could be limited in two ways. First, the line between these two categories of disorder policing programs can be blurred. For instance, order maintenance tactics can be implemented as the result of a problem‐oriented policing process and community concerns over disorderly social behaviors in public spaces. Second, the strategies within each of these broad categories can differ greatly depending on the targeted crime and disorder problem. An aggressive order maintenance program focused on the disorderly behaviors of violent gang members could include tactics that differ from those used in a program to control more general disorderly behavior of citizens. Future research testing the impacts of different disorder policing strategies on crime should do so with high‐quality research designs.

It is important to note that our systematic review was not designed to test the key theoretical propositions of the broken windows perspective on the links among disorder, fear, informal social control, and more serious crime in neighborhoods (Wilson & Kelling, 1982). Indeed, many of the effective policing disorder strategies reviewed here concentrate police action in crime hot spots. Deterrence and opportunity theories are usually applied to understand the crime control gains generated by hot spots policing (Braga & Weisburd, 2010; Nagin, 2013). From the standpoint of crime control and prevention, of course, the distinctions among deterrence, opportunity reduction, and broken windows are irrelevant—it only matters whether an intervention “works” by increasing public safety (for an argument to this effect, see Miles & Ludwig, 2007). From the standpoint of theory, on the other hand, these distinctions are of paramount importance and the time is ripe to develop a rigorous body of evaluation evidence to understand the mechanisms associated with successful disorder policing programs (see Weisburd et al., 2015).

It is also noteworthy that the results of this systematic review and meta‐analysis lend some credibility to the NYPD's claim that disorder policing was influential in reducing crime in New York City over the course of the 1990s. But explaining the city's crime drop over the last two decades remains a puzzling challenge to social scientists. As Rosenfeld et al. (2014) suggest, social scientists who study crime trends have not been satisfied by existing research that seeks to explain this phenomenon. Indeed, it is this lack of satisfaction that keeps the cottage industry of nonexperimental analyses of New York City crime trends alive. Given the complexities involved in modeling crime trends, we believe that no multivariate analysis will adequately settle this ongoing debate. While new experiments in New York City will not alone solve the city's crime drop puzzle, careful controlled evaluations could go a long way in settling the related debates on the crime control efficacy of policing disorder programs.

## ROLES AND RESPONSIBILITIES

A. A. B., B. C. W., and C. S. designed the original systematic review following established Campbell Collaboration conventions and protocols. With the assistance of Phyllis Schultze, C. S., and A. A. B. executed the varied search strategies to identify eligible studies. A. A. B., B. C. W., and C. S. selected eligible studies that fit the established criteria and coded the characteristics of the eligible studies. A. A. B., B. C. W., and C. S. calculated standardized mean effect sizes and executed the formal meta‐analyses. A. A. B., B. C. W., and C. S. wrote the narrative reviews for each eligible study. A. A. B., B. C. W., and C. S. collaborated closely on the writing of the literature review, methodology and analysis sections, results, and conclusion.

## Footnotes

1
https://www.campbellcollaboration.org/effect‐size‐calculato.html.

2 The landmark Kansas City Preventive Patrol Experiment had to be stopped and restarted three times before it was implemented properly; the patrol officers did not respect the boundaries of the treatment and control areas (Kelling, Pate, Dickman, & Brown, 1974). Likewise, the design of the Minneapolis Spouse Abuse Experiment was modified to a quasiexperiment when randomization could not be achieved because officers chose to arrest certain offenders on a nonrandom basis (Berk, Smyth, & Sherman, 1988).

3
*Q* = 426.119, df = 29, *p* < .05, *I* ^2^ = 93.194; *τ* ^2^ = 0.033, *SE* = 0.026, *τ* = 0.183.

4 Random effects models were used to estimate the overall standardized mean effect sizes. For the largest effect size meta‐analysis, *Q* = 531.829, df = 29, *p* < .05, *I* ^2^ = 94.527; *τ* ^2^ = 0.054, *SE* = 0.042, *τ* = 0.232. For the smallest effect size meta‐analysis, *Q* = 389.481, df = 29, *p* < .05, *I* ^2^ = 92.554; *τ* ^2^ = 0.039, *SE* = 0.032, *τ* = 0.198.

5 For the crime displacement and diffusion meta‐analysis, *Q* = 2843.833, df = 14, *p *< .05, *I* ^2^ = 99.508; *τ* ^2^ < 0.001, *SE* = 0.001, *τ* = 0.022.

6 We used a random effects model for this comparison. For the quasiexperiments, *Q* = 398.634, df = 22, *p *< .05, *I* ^2^ = 94.481; *τ* ^2^ = 0.042, *SE* = 0.037, *τ* = 0.205. For the randomized controlled trials, *Q *= 11.824, df = 6, *p* = .066, *I* ^2^ = 49.258; *τ* ^2^ = 0.005, *SE* = 0.006, *τ* = 0.067.

7 A random effects model was used because the within‐group effect size variation was determined to be heterogeneous for the two program types. For community problem‐oriented policing programs, *Q* = 405.668, df = 19, *p* < .05, *I* ^2^ = 95.316; *τ* ^2^ = 0.039, *SE* = 0.033, *τ* = 0.197. For increased policing programs, *Q* = 18.883, df = 9, *p* < .05, *I* ^2^ = 52.338; *τ* ^2^ = 0.011, *SE* = 0.011, *τ* = 0.104. The between group *Q* = 8.582, df = 1, *p* < .05.

## APPENDIX 1 LIST OF 120 EXPERTS CONTACTED DURING SEARCH PROCESS

1. David Bayley, University at Albany, SUNY

2. Gisela Bichler‐Robertson, California State University ‐ San Bernardino

3. Alfred Blumstein, Carnegie Mellon University

4. Rachel Boba Santos, Florida Atlantic University

5. Brenda Bond, Suffolk University

6. Kate Bowers, University College London

7. Gerben Bruinsma, Netherlands Institute for the Study of Crime and Law Enforcement

8. Rod Brunson, Rutgers University

9. Michael Buerger, Bowling Green State University

10. Timothy Bynum, Michigan State University

11. George Capowich, Loyola University, New Orleans

12. Steven Chermak, Michigan State University

13. Ronald V. Clarke, Rutgers University

14. Jacqueline Cohen, Carnegie Mellon University

15. James Coldren, Governors State University

16. Phillip Cook, Duke University

17. Gary Cordner, Kutztown University

18. Nicholas Corsaro, University of Cincinnati

19. David Curry, University of Missouri ‐ St. Louis

20. Robert Davis, RAND Corporation

21. Scott Decker, Arizona State University

22. John E. Eck, University of Cincinnati

23. Robin Engel, University of Cincinnati

24. Graham Farrell, Loughborough University

25. David Farrington, University of Cambridge

26. Brian Frost, American University

27. Patrick Gartin, Missouri State University

28. Andrew Giacomazzi, Boise State University

29. Herman Goldstein, University of Wisconsin

30. Peter Grabosky, Australian National University

31. Clifford Grammich, RAND Corporation

32. Jack Greene, Northeastern University

33. Joshua Hinkle, Georgia State University

34. Natalie Hipple, Michigan State University

35. Alexander Holsinger, University of Missouri ‐ Kansas City

36. Timothy Hope, Keele University

37. Joel Horowitz, Northwestern University

38. Shane Johnson, University College London

39. Charles Katz, Arizona State University

40. George Kelling, Rutgers University

41. David M. Kennedy, John Jay College of Criminal Justice

42. John Kilburn, Texas A&M International University

43. Mark A. R. Kleiman, University of California ‐ Los Angeles

44. David A. Klinger, University of Missouri, St. Louis

45. John Klofas, Rochester Institute of Technology

46. Johannes Knutsson, Norwegian Police University College

47. Christopher Koper, George Mason University

48. Janet Lauritsen, University of Missouri, St. Louis

49. Brian Lawton, George Mason University

50. Gloria Laycock, University College London

51. Steven Levitt, University of Chicago

52. Jens Ludwig, University of Chicago

53. Cynthia Lum, George Mason University

54. John MacDonald, University of Pennsylvania

55. Edward R. Maguire, American University

56. Peter Manning, Northeastern University

57. Stephen D. Mastrofski, George Mason University

58. Lorraine Mazerolle, University of Queensland

59. Jack McDevitt, Northeastern University

60. Edmund McGarrell, Michigan State University

61. Tracey Meares, Yale University

62. Mark H. Moore, Harvard University

63. Daniel Nagin, Carnegie Mellon University

64. Graeme Newman, University at Albany, State University of New York

65. Kenneth Novak, University of Missouri ‐ Kansas City

66. Andrew V. Papachristos, Yale University

67. John Pepper, University of Virginia

68. Ruth Peterson, Ohio State University

69. Anne Piehl, Rutgers University

70. Glenn L. Pierce, Northeastern University

71. Alex Piquero, University of Texas at Dallas

72. Jerry Ratcliffe, Temple University

73. Justin Ready, Arizona State University

74. George Rengert, Temple University

75. Peter Reuter, University of Maryland

76. Greg Ridgeway, RAND Corporation

77. Jan Roehl, Jan Roehl Consulting

78. Colin Rogers, University of Glamorgan

79. Dennis Rosenbaum, University of Illinois, Chicago

80. Richard Rosenfeld, University of Missouri, St. Louis

81. Rana Sampson, Community Policing Associates

82. Robert Sampson, Harvard University

83. David Schaefer, Arizona State University

84. Michael Scott, University of Wisconsin, Madison

85. Lawrence Sherman, University of Cambridge

86. Eli Silverman, John Jay College of Criminal Justice

87. Wesley Skogan, Northwestern University

88. Jerome Skolnick, New York University

89. Michael Smith, University of Texas ‐ E l Paso

90. William Sousa, University of Nevada, Las Vegas

91. William Spelman, University of Texas, Austin

92. Elaine B. Sharp, University of Kansas

93. Travis Taniguchi, Police Foundation

94. Bruce Taylor, University of Chicago

95. Ralph Taylor, Temple University

96. Cody Telep, George Mason University

97. Quint Thurman, Sul Ross State University

98. Nick Tilley, University College London

99. Marie Tillyer, University of Texas, San Antonio

100. George E. Tita, University of California, Irvine

101. Jeremy Travis, John Jay College of Criminal Justice

102. Michael Turner, University of North Carolina Charlotte

103. Tom Tyler, New York University

104. Craig Uchida, Justice and Security Strategies

105. Sean Varano, Roger Williams University

106. Joel Waldfogel, University of Minnesota

107. Samuel Walker, University of Nebraska, Omaha

108. Vincent Webb, Sam Houston State

109. David Weisburd, George Mason University

110. Deborah Lamm Weisel, North Carolina State University

111. Alexander Weiss, Northwestern University

112. Charles Wellford, University of Maryland

113. James Willis, George Mason University

114. Jeremy M. Wilson, Michigan State University

115. Jennifer Wood, Temple University

116. Daniel Woods, Police Executive Research Forum

117. Robert Worden, University at Albany, SUNY

118. Laura Wyckoff, University of Maryland

119. Sue‐Ming Yang, Georgia State University

120. Franklin Zimring, University of California ‐ Berkeley

## APPENDIX 2 DISORDER POLICING STUDY CODING INSTRUMENT

**Reference Information**

1. Document ID: __ __ __ __

2. Study author(s): _______________________________________________________________________________________________________________________________________

3. Study title: _____________________________________________________________________________________________________________________________________________

4. Publication type: ______

1. Book

2. Book chapter

3. Journal article (peer reviewed)

4. Thesis or doctoral dissertation

5. Government report (state/local)

6. Government report (federal)

7. Police department report

8. Technical report

9. Conference paper

10. Other (specify)) _____________________

5. Publication date (year): _____________________

6a. Journal Name: _______________________________________________________________________________________________________________________________________

6b. Journal Volume: _____________________

6c. Journal Issue: _____________________

7. Date range of research (when research was conducted):

Start: _____________________

Finish: _____________________

8. Source of funding for study: _________________________________________________________________________________________________________________________

9. Country of publication: ___________________

10. Date coded: _____________________

11. Coder's Initials: __ __ __ __

**Describing the Disorder Policing Intervention**

12a. Did the study formally identify the treatment as a disorder policing intervention?

1. Yes

2. No

12b. If No, what did the study call the intervention? ________________________________________________________________________________________________

13. What crime problem was targeted for the intervention? (Select all that apply)

1. Total crime

2. Violent crime

3. Property crime

4. Homicide

5. Sexual assault/rape

6. Robbery

7. Assault

8. Burglary

9. Larceny

10. Motor vehicle theft

11. Disorder

12. Other (specify) _______________________________________________________________________________________________________________________________________

14. What unit of analysis was used? (Select all that apply)

1. Crime hot spots (specific small places)

2. Police‐defined geographical unit (districts, precincts, etc.)

3. U.S. Census‐defined geographical unit (tracts, block groups, etc.)

4. Neighborhoods as defined by researchers

5. Other unit (specify) ___________________________________________________________________________________________________________________________________

15. What type of disorder policing intervention was implemented in the targeted areas?

(Select all that apply)

1. Community input on disorder problems

2. Increased misdemeanor arrests to control social disorder

3. Situational strategies to modify physical environment

4. Social service strategies to deal with disorderly persons (substance abusers,

homeless, mentally‐ill offenders, etc.)

5. Other (specify) ________________________________________________________________________________________________________________________________________

16. What did the evaluation indicate about the implementation of the response? _____________________

1. The response was implemented as planned or nearly so

2. The response was not implemented or implemented in a radically different way

than originally planned

3. Unclear/no process evaluation included

17. If the process evaluation indicated there were problems with implementation of the

response, describe these problems:

____________________________________________________________________________________________________________________________________________________________

____________________________________________________________________________________________________________________________________________________________

____________________________________________________________________________________________________________________________________________________________

18. Country where study was conducted: ___________________________

19. City (and state/province, if applicable) where study was conducted: ___________________________________________________________________________

**Methodology/Research design:**

20. Type of study: _____________________

1. Randomized experiment

2. Nonequivalent control group (quasi‐experimental)

3. Multiple time series (quasi‐experimental)

4. Other (specify) _____________________

21. How were study units allocated to treatment or comparison conditions?

1. Simple random allocation

2. Random allocation in pairs, blocks, or some other sophisticated technique

3. Simple descriptive matching

4. Sophisticated statistical matching (e.g. propensity scores)

5. Other (specify) _____________________

22. Explain how independent and extraneous variables were controlled so that it was possible to disentangle the impact of the intervention or how threats to internal validity were ruled out.

____________________________________________________________________________________________________________________________________________________________

____________________________________________________________________________________________________________________________________________________________

____________________________________________________________________________________________________________________________________________________________

____________________________________________________________________________________________________________________________________________________________

____________________________________________________________________________________________________________________________________________________________

23. Did the study measure spatial crime displacement and diffusion of crime control benefits?

1. Yes

2. No

24. Explain how the study measured spatial crime displacement and diffusion of crime control benefits.

____________________________________________________________________________________________________________________________________________________________

____________________________________________________________________________________________________________________________________________________________

____________________________________________________________________________________________________________________________________________________________

____________________________________________________________________________________________________________________________________________________________

____________________________________________________________________________________________________________________________________________________________

*The following questions refer to the units receiving treatment*:

25. Units receiving treatment: _____________________

1. Crime hot spots (specific small places)

2. Police‐defined geographical unit (districts, precincts, etc.)

3. U.S. Census‐defined geographical unit (tracts, block groups, etc.)

4. Neighborhoods as defined by researchers

5. Other unit (specify) _____________________

26. What is the exact unit receiving treatment?

____________________________________________________________________________________________________________________________________________________________

*The following question refers to the units not receiving treatment*

27. Units NOT receiving treatment: _____________________

1. Crime hot spots (specific small places)

2. Police‐defined geographical unit (districts, precincts, etc.)

3. U.S. Census‐defined geographical unit (tracts, block groups, etc.)

4. Neighborhoods as defined by researchers

5. Other unit (specify) _____________________

28. What were the casual hypotheses tested in this study?

____________________________________________________________________________________________________________________________________________________________

____________________________________________________________________________________________________________________________________________________________

____________________________________________________________________________________________________________________________________________________________

29. Please identify any theories from which the causal hypotheses were derived.

____________________________________________________________________________________________________________________________________________________________

____________________________________________________________________________________________________________________________________________________________

____________________________________________________________________________________________________________________________________________________________

***Outcomes reported** (Note that for each outcome, a separate coding sheet is required. This includes main effects outcomes as well as crime displacement and diffusion of crime control benefits outcomes)*

30. How many crime/alternative outcomes are reported in the study? _____________________

31. What is the specific outcome recorded on this coding sheet?

____________________________________________________________________________________________________________________________________________________________

32. Was it the primary outcome of the study? _____________________

1. Yes

2. No

3. Can't tell/researcher did not prioritize outcomes

33. Was this initially intended as an outcome of the study? _____________________

1. Yes

2. No (explain)

3. Can't tell

34. If no, explain why:

____________________________________________________________________________________________________________________________________________________________

____________________________________________________________________________________________________________________________________________________________

***Unit of analysis***

35. What was the unit of analysis for the research evaluation?

1. Crime hot spots (specific small places)

2. Police‐defined geographical unit (districts, precincts, etc.)

3. U.S. Census‐defined geographical unit (tracts, block groups, etc.)

4. Neighborhoods as defined by researchers

5. Other unit (specify) ___________________________________________________________________________________________________________________________________

36. How many units of analysis are there for the intervention in the study? ______________________________________________________________________

37. Did the researchers collect nested data within the unit of analysis?

1. Yes

2. No

***Dependent Variable***

38. What type of data was used to measure the outcome covered on this coding sheet?

1. Official data (from the police)

2. Researcher observations

3. Self‐report surveys

4. Other (specify) ________________________________________________________________________________________________________________________________________

39. If official data was used, what specific type(s) of data were used? (Select all that apply)

1. Calls for service (911 calls)/crime reports

2. Arrests

3. Incident reports

4. Level of citizen complaints

5. Other (specify)

6. N/A (official data not used)

7. Other (specify) ________________________________________________________________________________________________________________________________________

40. If researcher observations were used, what types of observations were taken? (Select all that apply)

1. Physical observations (e.g. observed urban blight, such as trash, graffiti)

2. Social observations (e.g. observed disorder, such as loitering, public drinking)

3. Other observations (specify)

4. N/A (researcher observations not used)

5. Other (specify) ________________________________________________________________________________________________________________________________________

41. If self‐report surveys were used, who was surveyed? (Select all that apply)

1. Residents/community members

2. Business owners

3. Elected officials

4. Government/social service agencies

5. Other (specify) ________________________________________________________________________________________________________________________________________

6. N/A (self‐report surveys not used)

42. Specifically identify the outcome covered on this coding sheet _________________________________________________________________________________

43. For the units of analysis in this study, what time periods were examined for the outcome covered on this coding sheet?

1. Yearly

2. Monthly

3. Weekly

4. Other researcher defined time periods (specify) ____________________________________________________________________________________________________

44. What was the length in time of the follow‐up period after the intervention?

____________________________________________________________________________________________________________________________________________________________

45. Did the researcher assess the quality of the data collected?

1. Yes

2. No

46a. Did the researcher(s) express any concerns over the quality of the data?

1. Yes

2. No

46b. If yes, explain

____________________________________________________________________________________________________________________________________________________________

____________________________________________________________________________________________________________________________________________________________

**Effect size/Reports of statistical significance**

***Dependent Measure Descriptors***

47. Statistical analysis design: _____________________

1. Pretest comparison

2. Post‐test comparison

3. Follow‐up comparison

4. N/A

*Sample Size*

48. Based on the unit of analysis for this outcome, what is the total sample size in the analysis?

____________________________________________________________________________________________________________________________________________________________

49. What is the total sample size of the treatment group (group that receives the response)?

____________________________________________________________________________________________________________________________________________________________

50. What is the total sample size of the control group (if applicable)? _____________________________________________________________________________

51a. Was attrition a problem in the analysis for this outcome?

1. Yes

2. No

51b. If attrition was a problem, provide details (e. g. how many cases were lost and why were they lost).

____________________________________________________________________________________________________________________________________________________________

____________________________________________________________________________________________________________________________________________________________

____________________________________________________________________________________________________________________________________________________________

52. What do the sample sizes above refer to?

1. Crime hot spots (specific small places)

2. Police‐defined geographical unit (districts, precincts, etc.)

3. U.S. Census‐defined geographical unit (tracts, block groups, etc.)

4. Neighborhoods as defined by researchers

5. Other unit (specify) ___________________________________________________________________________________________________________________________________

***Effect Size Data***

53. Raw difference favors (i.e. shows more success for):

1. Treatment group

2. Control group

3. Neither (exactly equal)

4. Cannot tell (or statistically insignificant report only)

54. Did a test of statistical significance indicate statistically significant differences between either the control and treatment groups or the pre and post tested treatment group? _____________________

1. Yes

2. No

3. Can't tell

4. N/A (no testing completed)

55. Was a standardized effect size reported?

1. Yes

2. No

56. If yes, what was the effect size? ___________________________________________________________________________________________________________________

57. If yes, page number where effect size data is found _____________________________________________________________________________________________

58. If no, is there data available to calculate an effect size?

1. Yes

2. No

59. Type of data effect size can be calculated from:

1. Means and standard deviations

2. *t*‐value or *F*‐value

3. Chi‐square (df=1)

4. Frequencies or proportions (dichotomous)

5. Frequencies or proportions (polychotomous)

6. Other (specify) ________________________________________________________________________________________________________________________________________

*Means and Standard Deviations*

60a. Treatment group mean. ____________________________________________________________________________________________________________________________

60b. Control group mean. _______________________________________________________________________________________________________________________________

61a. Treatment group standard deviation. _____________________________________________________________________________________________________________

61b. Control group standard deviation. ________________________________________________________________________________________________________________

*Proportions or frequencies*

62a. *n* of treatment group with a successful outcome. ________________________________________________________________________________________________

62b. *n* of control group with a successful outcome. ____________________________________________________________________________________________________

63a. Proportion of treatment group with a successful outcome. _____

63b. Proportion of treatment group with a successful outcome. _____

*Significance Tests*

64a. *t*‐value ______________________________________________________________________________________________________________________________________________

64b. *F*‐value ______________________________________________________________________________________________________________________________________________

64c. Chi‐square value (*df*=1) ____________________________________________________________________________________________________________________________

*Calculated Effect Size*

65a. Effect size __________________________________________________________________________________________________________________________________________

65b. Standard error of effect size _____________________

**Conclusions made by the author(s)**

*Note that the following questions refer to conclusions about the effectiveness of the intervention in regards to the current outcome being addressed on this coding sheet*.

66. Conclusion about the impact of the disorder policing intervention? _____________________

1. The authors conclude the program positively impacted crime/disorder

2. The authors conclude the problem did not positively impact crime/disorder

3. Unclear/no conclusion stated by authors

67. Did the assessment find evidence of a geographic displacement of crime? _____________________

1. Yes

2. No

3. Not tested

68. Did the assessment find evidence of other types of displacement of crime? _____________________

1. Yes. Please specify _____________________

2. No

3. Not tested

69. Did the assessment find evidence of a geographic diffusion of crime control benefits? _____________________

1. Yes

2. No

3. Not tested

70. Did the assessment find evidence of other types of diffusion of crime control benefits? _____________________

1. Yes. Please specify _____________________

2. No

3. Not tested

71. Did the author(s) conclude that the disorder policing intervention was beneficial? _____________________

1. Yes

2. No

3. Can't tell

72. Did the author(s) conclude there was a relationship between the disorder policing intervention and a reduction in crime? __________________

1. Yes

2. No

3. Can't tell

73. Who funded the intervention?

____________________________________________________________________________________________________________________________________________________________

____________________________________________________________________________________________________________________________________________________________

74. Who funded the evaluation research?

____________________________________________________________________________________________________________________________________________________________

____________________________________________________________________________________________________________________________________________________________

75a. Were the researchers independent evaluators?

1. Yes

2. No

75b. If no, explain the nature of the relationship:

____________________________________________________________________________________________________________________________________________________________

____________________________________________________________________________________________________________________________________________________________

76. Additional notes about conclusions:

____________________________________________________________________________________________________________________________________________________________

____________________________________________________________________________________________________________________________________________________________

77. Additional notes about study:

____________________________________________________________________________________________________________________________________________________________

____________________________________________________________________________________________________________________________________________________________
